# Preadmission morbidity and healthcare utilization among older adults with potentially avoidable hospitalizations: a Danish case–control study

**DOI:** 10.1007/s41999-023-00887-7

**Published:** 2023-11-28

**Authors:** Christine K. Schrøder, Eskild B. Kristiansen, Lone Flarup, Christian F. Christiansen, Reimar W. Thomsen, Pia K. Kristensen

**Affiliations:** 1 Department of Clinical Epidemiology, Aarhus University Hospital, Olof Palmes Allé 43-45, 8200 Aarhus N, Denmark; 2 Department of Orthopedic Surgery, Aarhus University Hospital, Palle Juul-Jensens, Boulevard 99, 8200 Aarhus N, Denmark; 3 Department of Clinical Medicine, Aarhus University, Palle Juul-Jensens Boulevard 82, 8200 Aarhus N, Denmark; 4 Strategisk Kvalitet, Koncern Kvalitet, Central Denmark Region, Viborg, Denmark

**Keywords:** Older adults, Preventable hospitalization, Ambulatory care sensitive conditions, Healthcare utilization

## Abstract

**Purpose:**

Examine preadmission diagnoses, medication use, and preadmission healthcare utilization among older adults prior to first potentially avoidable hospitalizations.

**Methods:**

A nationwide population-based case–control study using Danish healthcare data. All Danish adults aged ≥ 65 years who had a first potentially avoidable hospitalization from January 1995 through March 2019 (*n* = 725,939) were defined as cases, and 1:1 age- and sex-matched general population controls (*n* = 725,939). Preadmission morbidity and healthcare utilization were assessed based on a complete hospital diagnosis history within 10 years prior, and all medication use and healthcare contacts 1 year prior. Using log-binomial regression, we calculated adjusted prevalence ratios (PR) with 95% confidence intervals (CI).

**Results:**

Included cases and controls had a median age of 78 years and 59% were female. The burden of preadmission morbidity was higher among cases than controls. The strongest associations were observed for preadmission chronic lung disease (PR 3.8, CI 3.7–3.8), alcohol-related disease (PR 3.1, CI 3.0–3.2), chronic kidney disease (PR 2.4, CI 2.4–2.5), psychiatric disease (PR 2.2, CI 2.2–2.3), heart failure (PR 2.2, CI 2.2–2.3), and previous hospital contacts with infections (PR 2.2, CI 2.2–2.3). A high and accelerating number of healthcare contacts was observed during the months preceding the potentially avoidable hospitalization (having over 5 GP contacts 1 month prior, PR 3.0, CI 3.0–3.0).

**Conclusion:**

A high number of healthcare contacts and preadmission morbidity and medication use, especially chronic lung, heart, and kidney disease, alcohol-related or psychiatric disease including dementia, and previous infections are strongly associated with potentially avoidable hospitalizations.

**Supplementary Information:**

The online version contains supplementary material available at 10.1007/s41999-023-00887-7.

## Key summary points

**Aim:**

Examine preadmission diagnoses, medication use, and preadmission healthcare utilization among older adults prior to a first potentially avoidable hospitalization.

**Findings:**

Our analysis revealed that preadmission morbidity, medication use, and a high and accelerating number of healthcare contacts are associated with potentially avoidable hospitalizations.

**Message:**

Preadmission morbidity and a high number of contacts with general practitioners are associated with potentially avoidable hospitalizations, which highlights the need for tailored clinical initiatives for older adults.

**Supplementary Information:**

The online version contains supplementary material available at 10.1007/s41999-023-00887-7.

## Introduction

Globally, healthcare systems are facing unprecedented challenges with shrinking budgets, a mismatch between workforce supply and system demand, increasing life expectancy, and with that a concomitant increase in the prevalence of chronic diseases, multimorbidity, and frailty [1–3]. It is a prerequisite when trying to meet these challenges, that the healthcare system must have a greater focus on the prevention of hospitalizations for older adults.

Potentially avoidable hospitalizations are acute hospital admissions for a set of conditions, referred to as ambulatory care sensitive conditions (ACSC) [4–7]. There are various definitions of which conditions are classified as ACSCs internationally, but frequent conditions include respiratory tract infections, dehydration, gastroenteritis, urinary tract infections (UTIs), and anemia [4, 8]. Hospitalizations for ACSCs are considered to be preventable, given timely and adequate care in the community-based healthcare setting including regular contacts with primary care providers such as general practitioners (GPs) who can prevent, control, and/or manage many diseases. ACSCs can, therefore, be used as an indicator of the accessibility and quality of primary care [4, 9, 10].

Previous observational studies have shown that older adults, males, and persons with low socioeconomic status have higher rates of potentially avoidable hospitalizations [11–14]. Other studies have indicated that preadmission morbidity, medication use, and preadmission healthcare utilization are important markers of avoidable hospitalizations, though, the majority of these studies have been limited by small size, hampering their ability to assess the impact of individual diseases [15–21]. Furthermore, previous studies often examined selected subpopulations such as residents in nursing homes and assisted living facilities [15–21].

To reduce potentially avoidable hospitalizations among older adults in the general population, it is important to know the exact risk markers in population-based settings. Population-based studies can also provide information about which underlying factors should be adjusted for when using rates of potentially avoidable hospitalizations as a quality measure in healthcare systems [11]. We, therefore, conducted a nationwide population-based case–control study in Denmark to examine preadmission diagnoses, medication use, and preadmission healthcare utilization among older adults prior to a first potentially avoidable hospitalization. We evaluated potentially avoidable hospitalizations overall, and specific admission diagnoses including fractures, respiratory tract infections, dehydration, urinary tract infections (UTIs), obstipation, gastroenteritis, anemia, admission for social reasons, and pressure ulcers.

## Methods

We conducted our case–control study with density sampling within a source of all Danish citizens aged 65 or older in the period between January 1st, 1995 and March 31st 2019 [22]. We used data from the Civil Registration System [23], the Danish National Patient Registry [24], the Danish National Prescription Registry [25], the Psychiatric Central Research Register [26], and the National Health Insurance Service Registry [27]. The study was reported to the Danish Data Protection Agency by registration at Aarhus University (record number: AU-2016-051-000001, sequential number 608). According to Danish law, an ethics committee approval is not required for registry-based studies.

### Data sources

The Civil Registration System was established in 1968. It is an administrative registry containing complete data on the date of birth, sex, sequential dates of migration, and vital status of every resident in Denmark. All Danish inhabitants have a unique civil registration number, which is used in all healthcare contacts and allows unambiguous linkage between the healthcare registries. The Civil Registration System is updated daily [23].

The Danish National Patient Registry was established in 1977 and contains information on all somatic in-patient hospitalizations in Danish public hospitals. Outpatient visits, emergency room visits, and psychiatric inpatients have been included since 1995. Patients are registered with primary and secondary diagnoses according to the International Classification of Diseases (ICD), with the 8^th^ revision from 1977 through 1993 and the 10th revision hereafter [24].

The Danish National Prescription Registry contains information for all prescriptions for reimbursed medicine dispensed by any community pharmacies in Denmark since 2004. The data are recorded according to the Anatomical Therapeutic Chemical classification system (ACT codes) and include the date of the redemption, number of pills, dose, number of defined daily doses, name and type of drug according to purchase, and the defined daily dose (DDD) [25].

The Psychiatric Central Research Register comprises persons who have been in touch with the psychiatric treatment systems, including district psychiatry, outpatient, and emergency services. In 1995, the register became a part of the National Patient Registry [26].

The National Health Insurance Service Registry holds information on contacts with healthcare professionals, such as GP and type of consultation (e.g. ordinary consultations, home visits, telephone consultations, and electronic consultations), services provided, and laboratory tests performed [27].

### Patients with potentially avoidable hospitalizations

From the Danish National Patient Registry, we identified patients aged 65 years or older with potentially avoidable hospitalizations from January 1st, 1995 to March 31st, 2019 (*n* = 725,939) defined as cases. In the Danish health system, potentially avoidable hospitalizations include acute hospital admissions among citizens aged 65 years or older with a primary diagnosis of one of the following nine conditions: fractures (ICD-10: S02, S12, S22, S32, S42, S52, S62, S72, S82, S92), respiratory tract infections (J12, J13, J14, J15, J18, J20, J21, J22, J40, J41, J42, J43, J44, J45, J46, J47), dehydration (E869), UTIs (N30, except N303 and N304), obstipation (K590), gastroenteritis (A09), anaemia due to nutrition (D50, D51, D52, D53), admission for social causes (Z59, Z74, Z75), and pressure ulcers (L89). The definition of potentially avoidable hospitalizations in Denmark is provided by the Danish Ministry of Health and has previously been used in Danish studies [14, 28].

### Matched controls from the background population

**Fig. 1:**
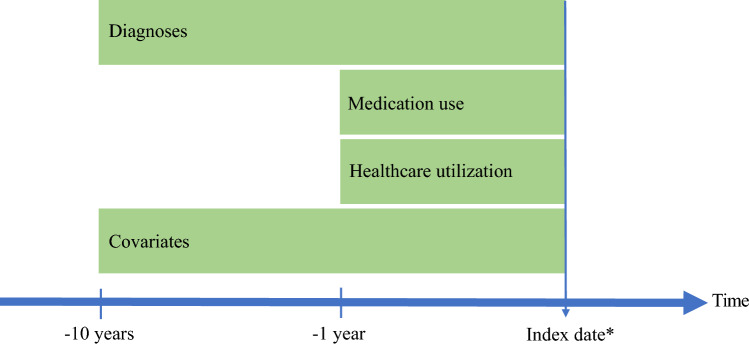
Visualization of the design used in the study. * Patient admitted with one of the nine ACSCs after having turned 65 years old. On the index date, a matched control person is selected with the same sex and age

At the time of the admission for each patient with a potentially avoidable hospitalization (the index date), we used the Civil Registration System to identify a control from the background population who had not had a previous primary diagnosis of potentially avoidable hospitalization by that time. The control from the background population was selected among those source population members of the same sex and year of birth as the cases. When examining potentially avoidable hospitalizations due to the nine potentially avoidable hospitalization diagnoses separately, the controls were selected among those with no such prior in-patient primary diagnosis of the potentially avoidable hospitalizations (i.e. a control for a case admitted with a fracture could not have a prior admission with a primary diagnosis of fractures, etc.) (Fig. 1). We sampled with replacement, that is, a person from the background population could act as a matched control to several of the cases.

### Preadmission morbidity and healthcare utilization

We examined previous diagnoses (somatic and psychiatric) and medication use prior to potentially avoidable hospitalizations. The diagnoses were assessed from ICD-codes for primary diagnoses from all hospital contacts 10 years prior to a preventable hospitalization and medication within 1 year (12 months) prior. Furthermore, we examined preadmission GP healthcare utilization (number of contacts, type of contact, and tests performed) 12, 3, and 1 months prior to admission. Additionally, the study comprised several descriptive variables including sociodemographic characteristics of the cases and controls [sex, age, and Charlson Comorbidity Index (CCI)] and clinical characteristics of the cases [time for admission, and details regarding the admission including surgical procedure and length of stay (LOS)] (Fig. 1).

### Statistical analysis

We tabulated the prevalence of the sociodemographic and clinical characteristics of the cases and compared them to the matched controls. We then calculated the prevalence ratios with 95% confidence intervals (95% CI) for the association between preadmission morbidity/healthcare utilization and potentially avoidable hospitalizations using a log-binomial regression model. We examined the association overall and hereafter made nine additional analyses to examine the association separately for the different potentially avoidable hospitalization diagnoses. The age- and sex-matched PRs were further adjusted for age and sex and then further adjusted for CCI. When the log-binomial model failed to converge, results were approximated by the Robust Poisson regression method. Data were analysed using SAS statistical software version 9.4 (SAS Institute Inc.).

## Results

### Sociodemographic and clinical characteristics

The study included 725,939 patients aged 65 years or older with potentially avoidable hospitalizations from 1995 to 2019 and 725,939 age- and sex-matched controls (Table 1). The cases and the controls had a median age of 78 years and 59% were female. The admission was most likely during the daytime, and the median LOS was 5 days. Compared to the controls the cases had a higher CCI score and more often lived alone.

**Table 1:** Differences in baseline characteristics between the cases and the sex- and age-matched controls

	Cases		Age- and sex-matched controls	
	*n*	(%)	*n*	(%)
All	725,939	(100)	725,939	(100)
Sex				
Female	429,370	(59.2)	429,370	(59.2)
Male	296,569	(40.9)	296,569	(40.9)
Age (median)	78.18			
65–74 years	273,776	(37.7)	274,037	(37.8)
75–84 years	279,686	(38.6)	279,708	(38.5)
≥ 85 years	172,477	(23.8)	172,194	(23.7)
Cohabiting				
Living alone	389,408	(53.6)	358,025	(49.3)
Divorced	86,532	(11.9)	65,644	(9.0)
Widow	292,940	(40.4)	278,820	(38.4)
Married	295,466	(40.7)	338,598	(46.6)
Missing	38,619	(5.3)	28,846	(4.0)
Charlson comorbidity index (CCI)				
CCI = 0	358,635	(49.4)	502,505	(69.2)
CCI = 1	165,393	(22.8)	111,574	(15.4)
CCI = 2	118,346	(16.3)	80,310	(11.1)
CCI ≥ 3	83,565	(11.5)	31,550	(4.4)
Time for admission				
Day	333,865	(46.0)	–	–
Evening	299,251	(41.2)	–	–
Night	75,527	(10.4)	–	–
Missing	17,296	(2.4)	–	–
Details regarding admission				
Surgical procedure	203,377	(28.0)	–	–
Length of stay (median), days*	5,581,768 (5)	–	–	–

*Total number of hospital nights

### Diagnoses and medications prior to potentially avoidable hospitalizations

In general, all preadmission diagnoses and medications were more prevalent among the cases compared to the controls (Table 2). The strongest association was observed for diagnoses and medications for chronic lung disease, e.g. previous chronic lung disease diagnoses (8.2% vs. 2.2%, PR: 3.8; 95% CI 3.7–3.8), asthma/COPD medication use (23.2% vs. 9.6%, PR: 2.5; 95% CI 2.4–2.5), and oral steroids (15.0% vs. 6.2%, PR: 2.4; 95% CI 2.4–2.5); for previous alcohol-related disease (1.9% vs. 0.6%, PR: 3.1; 95% CI 3.0–3.2); for chronic kidney disease diagnosis (2.6% vs. 1.1%, PR: 2.4; 95% CI 2.4–2.5), and for heart failure diagnosis (5.6% vs. 2.5%, PR: 2.2; 95% CI 2.2–2.3);. Furthermore, diagnoses and medications for psychiatric disease, e.g. previous psychiatric diagnoses (1.2% vs. 0.5%, PR: 2.2; 95% CI 2.2–2.3), use of antipsychotics (8.0% vs. 4.0%, PR: 2.0; 95% CI 2.0–2.0), and dementia diagnosis (2.9% vs. 1.3%, PR: 2.1; 95% CI 2.1–2.2) yielded a PR of 2 or higher. Moreover, previous hospital contact with infections had a strong association as well (12.0% vs. 5.4%, PR: 2.2; 95% CI 2.2–2.3). Besides this, several other psychiatric medications (including any nervous system, psycholeptics, and antidepressant drugs) and antibiotics (including any and systemic antibiotics) had an absolute difference between cases and controls of 11% to 20% and yielded a PR of 1.4 or above (Table 2).

**Table 2:** Diagnoses and medication use prior to preventable hospitalizations

	Cases		Controls		Unadjusted PR		Adjusted PR*		Adjusted PR†	
	*n*	(%)	*n*	(%)	PR	(95% CI)	PR	(95% CI)	PR	(95% CI)
Psychiatric disease										
D. of psychiatric disease‡	8,681	(1.2)	3,886	(0.5)	2.2	(2.2–2.3)	2.2	(2.2–2.3)	2.1	(2.0–2.2)
Any nervous system drugs	499,020	(68.7)	357,371	(49.2)	1.4	(1.4–1.4)	1.4	(1.4–1.4)	1.3	(1.3–1.3)
Psycholeptic drugs	272,352	(37.5)	190,202	(26.2)	1.4	(1.4–1.4)	1.4	(1.4–1.4)	1.4	(1.4–1.4)
Antipsychotics	58,233	(8.0)	28,971	(4.0)	2.0	(2.0–2.0)	2.0	(2.0–2.0)	2.0	(1.9–2.0)
Anxiolytics	128,168	(17.7)	83,527	(11.5)	1.5	(1.5–1.6)	1.5	(1.5–1.5)	1.5	(1.5–1.5)
Hypnotics and sedatives	167,374	(23.1)	118,565	(16.3)	1.4	(1.4–1.4)	1.4	(1.4–1.4)	1.3	(1.3–1.3)
Benzodiazepine derivatives	125,279	(17.3)	81,709	(11.3)	1.5	(1.5–1.6)	1.5	(1.5–1.5)	1.5	(1.5–1.5)
Antidepressant drugs	160,409	(22.1)	85,136	(11.7)	1.9	(1.9–1.9)	1.9	(1.9–1.9)	1.7	(1.7–1.7)
Dementia										
D. of dementia	20,673	(2.9)	9,643	(1.3)	2.1	(2.1–2.2)	2.1	(2.1–2.2)	1.7	(1.6–1.7)
Anti-dementia drugs	17,633	(2.4)	9,525	(1.3)	1.9	(1.8–1.9)	1.9	(1.8–1.9)	1.7	(1.7–1.8)
Cancer										
D. of cancer	121,443	(16.7)	70,189	(9.7)	1.7	(1.7–1.8)	1.7	(1.7–1.8)	1.0	(1.0–1.0)
Cardiovascular disease										
Any cardiovascular diagnosis	296,678	(40.9)	210,702	(29.0)	1.4	(1.4–1.4)	1.4	(1.4–1.4)	1.2	(1.2–1.2)
Ischemic heart disease	89,327	(12.3)	63,333	(8.7)	1.4	(1.4–1.4)	1.4	(1.4–1.4)	1.2	(1.2–1.2)
Heart failure	40,352	(5.6)	18,165	(2.5)	2.2	(2.2–2.3)	2.2	(2.2–2.3)	1.5	(1.5–1.5)
Heart valve disease	21,626	(3.0)	14,899	(2.1)	1.5	(1.4–1.5)	1.5	(1.4–1.5)	1.3	(1.2–1.3)
Atrial fibrillation	57,085	(7.9)	37,600	(5.2)	1.5	(1.5–1.5)	1.5	(1.5–1.5)	1.4	(1.3–1.4)
Peripheral artery disease	47,943	(6.6)	26,290	(3.6)	1.8	(1.8–1.9)	1.8	(1.8–1.9)	1.2	(1.1–1.2)
Deep vein thrombosis	11,915	(1.6)	7,666	(1.1)	1.6	(1.5–1.6)	1.6	(1.5–1.6)	1.4	(1.3–1.4)
Hypertension	42,342	(5.8)	31,742	(4.4)	1.3	(1.3–1.4)	1.3	(1.3–1.4)	1.3	(1.1–1.2)
Any cardiovascular medication	512,201	(70.6)	451,907	(62.3)	1.1	(1.1–1.1)	1.1	(1.1–1.1)	1.0	(1.0–1.0)
Antihypertensives§	425,208	(58.6)	384,306	(52.9)	1.1	(1.1–1.1)	1.1	(1.1–1.1)	1.1	(1.1–1.1)
Agents acting on RAS	209,565	(28.9)	192,634	(6.5)	1.1	(1.1–1.1)	1.1	(1.9–1.1)	1.0	(1.0–1.0)
Other antihypertensives	262,398	(36.2)	235,288	(32.4)	1.1	(1.1–1.1)	1.1	(1.1–1.1)	1.0	(1.0–1.1)
Loop diuretics	192,297	(26.5)	105,059	(14.5)	1.8	(1.8–1.8)	1.8	(1.8–1.8)	1.6	(1.6–1.6)
Digoxin	66,837	(9.2)	43,446	(6.0)	1.5	(1.5–1.6)	1.5	(1.5–1.5)	1.4	(1.4–1.4)
Nitrates	68,778	(9.5)	48,727	(6.7)	1.4	(1.4–1.4)	1.4	(1.4–1.4)	1.3	(1.3–1.3)
Lipid modifying agents	150,819	(20.8)	139,168	(19.2)	1.1	(1.1–1.1)	1.1	(1.1–1.1)	1.0	(0.9–1.0)
NOAC||	15,593	(2.2)	9,404	(1.3)	1.7	(1.6–1.7)	1.7	(1.6–1.7)	1.4	(1.4–1.5)
Platelet-aggregation prophylaxis	257,218	(35.4)	204,946	(28.2)	1.3	(1.3–1.3)	1.2	(1.2–1.3)	1.1	(1.1–1.1)
Vitamin K antagonists	52,262	(7.2)	35,361	(4.9)	1.5	(1.5–1.5)	1.5	(1.5–1.5)	1.3	(1.3–1.3)
Cerebrovascular disease										
Any cerebrovascular disease	73,695	(10.2)	40,723	(5.6)	1.8	(1.8–1.8)	1.8	(1.8–1.8)	1.3	(1.3–1.3)
D. of stroke and TIA	78,172	(10.8)	47,301	(6.5)	1.7	(1.6–1.7)	1.7	(1.6–1.7)	1.2	(1.2–1.2)
Diabetes										
D. of diabetes	41,766	(5.8)	23,452	(3.2)	1.8	(1.8–1.8)	1.8	(1.8–1.8)	1.0	(1.0–1.0)
Antidiabetics	78,023	(10.8)	55,132	(7.6)	1.4	(1.4–1.4)	1.4	(1.4–1.4)	1.1	(1.1–1.1)
Osteoporosis										
D. of osteoporosis	33,787	(4.7)	20,289	(2.8)	1.7	(1.6–1.7)	1.7	(1.6–1.7)	1.6	(1.5–1.6)
Chronic lung disease										
D. of chronic lung disease	59,827	(8.2)	16,042	(2.2)	3.7	(3.7–3.8)	3.8	(3.7–3.8)	2.6	(2.5–2.6)
Asthma/COPD medication	168,683	(23.2)	69,419	(9.6)	2.4	(2.4–2.5)	2.5	(2.4–2.5)	2.3	(2.3–2.3)
Oral steroids	108,605	(15.0)	44,938	(6.2)	2.4	(2.4–2.4)	2.4	(2.4–2.5)	2.1	(2.1–2.2)
Alcoholism-related disease										
D. of alcoholism-related disease	13,995	(1.9)	4,514	(0.6)	3.1	(3.0–3.2)	3.1	(3.0–3.2)	2.6	(2.5–2.7)
Nutrition-related disease										
D. of obesity	2,562	(0.4)	1,495	(0.2)	1.7	(1.6–1.8)	1.7	(1.6–1.8)	1.4	(1.3–1.5)
D. of undernutrition	3,728	(0.5)	2,045	(0.3)	1.8	(1.7–1.9)	1.8	(1.7–1.9)	1.5	(1.4–1.6)
Hypertrophia prostatae										
D. of hypertrophia prostatae	32,617	(4.5)	26,970	(3.7)	1.2	(1.2–1.2)	1.2	(1.2–1.2)	1.1	(1.1–1.1)
Connective tissue disease										
D. of connective tissue disease	25,489	(3.5)	15,836	(2.2)	1.6	(1.6–1.6)	1.6	(1.6–1.6)	1.1	(1.1–1.1)
Peptic ulcer disease										
D. of peptic ulcer	29,587	(4.1)	16,177	(2.2)	1.8	(1.8–1.9)	1.8	(1.8–1.9)	1.2	(1.2–1.2)
Gastric acid-related drugs	209,204	(28.8)	131,254	(18.1)	1.6	(1.6–1.6)	1.6	(1.6–1.6)	1.4	(1.4–1.4)
Low vision or blindness										
D. of low vision or blindness	1,307	(0.2)	943	(0.1)	1.4	(1.3–1.5)	1.4	(1.3–1.5)	1.2	(1.1–1.3)
Hypercholesterolemia										
D. of hypercholesterolemia	3,376	(0.5)	3,066	(0.4)	1.1	(1.1,1.2)	1.1	(1.1,1.2)	0.9	(0.9,1.0)
Chronic kidney disease										
D. of chronic kidney disease	18,636	(2.6)	7,708	(1.1)	2.4	(2.4–2.5)	2.4	(2.4–2.5)	1.2	(1.2–1.2)
Infections										
Any antibiotic	424,104	(58.4)	288,028	(39.7)	1.5	(1.5–1.5)	1.5	(1.5–1.5)	1.4	(1.4–1.4)
Any D. of infections	87,018	(12.0)	38,966	(5.4)	2.2	(2.2–2.3)	2.2	(2.2–2.3)	1.9	(1.9–1.9)
Antibiotic systemic	383,453	(52.8)	240,957	(33.2)	1.6	(1.6–1.6)	1.6	(1.6–1.6)	1.5	(1.5–1.5)
D. of respiratory tract infection	40,129	(5.5)	14,116	(1.9)	2.8	(2.8–2.9)	2.9	(2.8–2.9)	2.4	(2.4–2.5)
D. of skin infections	27,365	(3.8)	16,317	(2.3)	1.7	(1.7–1.7)	1.7	(1.7–1.7)	1.4	(1.4–1.5)
Antibiotic topical	42,457	(5.9)	33,414	(4.6)	1.3	(1.3–1.3)	1.3	(1.3–1.3)	1.2	(1.2–1.2)
D. of sepsis	18,879	(2.6)	6,141	(0.9)	3.1	(3.0–3.2)	3.1	(3.0–3.2)	2.4	(2.3–2.5)
D. of intestinal infectious disease	15,629	(2.2)	9,641	(1.3)	1.6	(1.6–1.7)	1.6	(1.6–1.7)	1.5	(1.4–1.5)
Antibiotic intestinale	13,670	(1.9)	4,435	(0.6)	3.1	(3.0–3.2)	3.1	(3.0–3.2)	2.5	(2.5–2.7)
D. of urinary tract infection	27,199	(3.8)	11,817	(1.6)	2.3	(2.3–2.4)	2.3	(2.3–2.4)	2.0	(1.9–2.0)
Symptoms not classified										
Symptoms not classified	87,019	(12.0)	47,433	(6.5)	1.8	(1.8–1.9)	1.8	(1.8–1.9)	1.6	(1.6–1.6)
Observation										
D. observation	301,602	(41.6)	206,368	(28.4)	1.5	(1.5–1.5)	1.5	(1.5–1.5)	1.3	(1.2–1.3)

All diagnoses are identified with a lookback period of 10 years from the index date and all medication use is identified with a lookback period of 12 months from the index date

*Sex- and age-adjusted PR, †Sex-, age-, and CCI-adjusted PR, ‡Diagnosis of psychoses, schizophrenia, affective, and personality disorders, §Loop diuretics not included, ||Dabigatran, rivaroxaban, apixaban, and edoxaban

*D* Diagnosis, *PR* Prevalence ratio, *CI* Confidence interval, *CCI* Charlson Comorbidity Index, *RAS* Renin angiotensin system, *NOAC* Novel oral anticoagulants, *TIA* Transient ischemic attack

Other modestly strong associated diagnoses and medications (i.e. PR of 1.5–2.0) included peptic ulcer diagnosis and gastric acid-related drugs, any cerebrovascular diagnoses, diabetes diagnosis, cancer diagnosis, nutrition-related diagnoses, connective tissue disease diagnosis, and osteoporosis diagnosis. Among these predictors, gastric acid-related drugs had an absolute difference between cases and controls of 11% as opposed to nutrition-related diagnoses, which had an absolute difference of < 1% between cases and controls (Table 2).

Cardiovascular diagnoses and their treatment overall only yielded PRs of 1.4 and 1.1. However, PRs were higher (1.5 to 2.2) for several individual cardiovascular diagnoses, including diagnoses of heart failure, peripheral arterial disease, deep vein thrombosis, and atrial fibrillation, and were also clearly higher for medications, including loop diuretics, digoxin, and novel oral anticoagulants (NOAC). Furthermore, cardiovascular diagnoses and their treatment overall had an absolute difference of 12% and 8% between cases and controls but varied when examining the individual diagnoses and medications (Table 2).

When we further adjusted for the overall comorbidity burden estimated by CCI, PRs for diagnoses and medications declined considerably. However, a PR of around 2 or higher after CCI adjustment was still observed for psychiatric diagnoses, chronic lung disease diagnosis and medications, alcohol-related disease, and previous respiratory tract infections and sepsis. In contrast, a diagnosis of cancer and diabetes per se was no longer materially associated with avoidable hospitalization when adjusted for CCI (Table 2). Forest plots with PRs can be found in Appendix 1. In general, many of the strong associations were robust when stratifying for the nine individual potentially avoidable hospitalization diagnoses (Supplementary Figs. 1, 3, 5, 7, 9, 11, 13, 15, and 17).

### Healthcare utilization prior to potentially avoidable hospitalizations

The cases had a higher number of contacts with a GP than controls, both within 12, 3, and 1 months prior to their admission (Table 3, 4, 5). The vast majority, i.e. 89% of the cases, had over 5 GP contacts during the last 12 months, compared with 77% of the controls, corresponding to a PR of 1.2 (95% CI 1.2–1.2). The prevalence of GP contacts was successively higher in the months up to the potentially avoidable hospitalization. Having over 5 GP contacts within the last month before hospitalization was associated with a PR of 3.0 (95% CI 3.0–3.0) in cases versus controls (Table 5). In accordance with this, having no preadmission GP contacts was less prevalent among cases than controls (PR for no GP contact 12 months prior of 0.4, 3 months prior of 0.4, and 1 month prior of 0.5). Among cases, we observed more than double the PRs for GP contact types including home visits, social medicine-related consultations, and reimbursement for chronically or terminally ill patients. Furthermore, conversational therapy and recent tests including microbiological culture, urine, Strep-A, C-reactive protein, and hemoglobin tests were prevalent. Of note, not all types of GP services were risk markers of avoidable hospitalizations. Thus, preadmission GP out-of-hours contacts, preventive GP consultations and services, influenza vaccines, and cytology tests, were all less likely among cases than among the matched controls (Table 3, 4, 5). Forest plots with PRs can be found in Appendix 1. The findings of a high and accelerating number of preadmission contacts with a GP in the months prior to their hospitalization were generally robust when stratifying for the nine individual potentially avoidable hospitalization diagnoses (Supplementary Figs. 2, 4, 6, 8, 10, 12, 14, 16, and 18).

**Table 3:** Healthcare utilization 12 months prior to preventable hospitalizations

	Cases		Controls		Unadjusted PR		Adjusted PR*		Adjusted PR†	
	*n*	(%)	*n*	(%)	PR	(95% CI)	PR	(95% CI)	PR	(95% CI)
ES consultation or home visit	643,314	(88.6)	612,679	(84.4)	1.0	(1.0–1.0)	1.0	(1.0–1.0)	1.0	(1.0–1.0)
GP consultation or home visit	675,140	(93.0)	632,255	(87.1)	1.0	(1.0–1.0)	1.0	(1.0–1.0)	1.0	(1.0–1.0)
No contacts	19,984	(2.8)	53,222	(7.3)	0.4	(0.4–0.4)	0.4	(0.4–0.4)	0.5	(0.5–0.5)
1–4 contacts	60,031	(8.3)	116,433	(16.0)	0.5	(0.5–0.5)	0.5	(0.5–0.5)	0.6	(0.6–0.6)
≥ 5 contacts	645,924	(89.0)	556,284	(76.6)	1.2	(1.2–1.2)	1.2	(1.2–1.2)	1.1	(1.1–1.1)
Home visits	361	(0.1)	209	(0.0)	1.4	(1.2–1.7)	1.4	(1.2–1.7)	1.4	(1.1–1.7)
Preventive consultations/services	38,849	(5.4)	39,208	(5.4)	0.9	(0.9–0.9)	0.9	(0.9–0.9)	0.9	(0.9–0.9)
Social-medicine-related	69,812	(9.6)	29,483	(4.1)	2.2	(2.2–2.3)	2.2	(2.2–2.2)	2.1	(2.0–2.1)
GP conversational therapy	12,804	(1.8)	7,884	(1.1)	1.5	(1.5–1.6)	1.5	(1.5–1.6)	1.4	(1.4–1.4)
Reimbursement for chronically ill	5,742	(0.8)	1,859	(0.3)	2.9	(2.8–3.1)	2.9	(2.7–3.0)	2.3	(2.2–2.5)
Reimbursement for terminally ill	1,767	(0.2)	207	(0.0)	8.0	(7.0–9.3)	8.0	(6.9–9.2)	4.4	(3.8–5.1)
Dementia diagnosis and treatment	1,260	(0.2)	865	(0.1)	1.4	(1.3–1.5)	1.4	(1.3–1.5)	1.4	(1.3–1.5)
Influenza vaccine	207,437	(28.6)	195,725	(27.0)	1.0	(1.0–1.0)	1.0	(1.0–1.0)	1.0	(1.0–1.0)
Undergoing test at the GP										
Microbiological culture	100,403	(13.8)	66,506	(9.2)	1.4	(1.4–1.4)	1.4	(1.4–1.4)	1.4	(1.3–1.4)
Cytology test	6,636	(0.9)	8,580	(1.2)	0.7	(0.7–0.8)	0.7	(0.7–0.7)	0.7	(0.7–0.8)
Urine test	215,857	(29.7)	165,560	(22.8)	1.2	(1.2–1.2)	1.2	(1.2–1.2)	1.2	(1.2–1.2)
Strep-A test	12,494	(1.7)	11,009	(1.5)	1.1	(1.0–1.1)	1.1	(1.0–1.1)	1.0	(1.0–1.1)
Any blood test	275,958	(38.0)	250,496	(34.5)	1.0	(1.0–1.0)	1.0	(1.0–1.0)	1.0	(1.0–1.0)
C-reactive protein	136,879	(18.9)	91,131	(12.6)	1.4	(1.4–1.4)	1.4	(1.4–1.4)	1.3	(1.3–1.3)
Creatinine	8,462	(1.2)	7,233	(1.0)	1.1	(1.1–1.1)	1.1	(1.1–1.1)	1.1	(1.1–1.1)
Hemoglobin	190,260	(26.2)	152,886	(21.1)	1.2	(1.2–1.2)	1.2	(1.2–1.2)	1.1	(1.1–1.1)
Glucose	156,472	(21.6)	143,161	(19.7)	1.0	(1.0–1.0)	1.0	(1.0–1.0)	1.0	(1.0–1.0)
INR	28,773	(4.0)	20,668	(2.9)	1.3	(1.3–1.3)	1.3	(1.3–1.3)	1.2	(1.2–1.2)

*Sex- and age-adjusted PR, †Sex-, age-, and CCI-adjusted PR

*PR* Prevalence ratio, *CI* Confidence interval, *CCI* Charlson Comorbidity Index, *ES* Doctor from the emergency service, *GP* General practitioner, *INR* International normalized ratio

**Table 4:** Healthcare utilization 3 months prior to preventable hospitalizations

	Cases		Controls		Unadjusted PR		Adjusted PR*		Adjusted PR†	
	*n*	(%)	*n*	(%)	PR	(95% CI)	PR	(95% CI)	PR	(95% CI)
ES consultation or home visit	494,460	(68.1)	407,185	(56.1)	1.0	(1.0–1.0)	1.0	(1.0–1.0)	1.0	(1.0–1.0)
GP consultation or home visit	542,476	(74.7)	429,289	(59.1)	1.0	(1.0–1.0)	1.0	(1.0–1.0)	1.0	(1.0–1.0)
No contacts	74,247	(10.2)	185,322	(25.5)	0.4	(0.4–0.4)	0.4	(0.4–0.4)	0.5	(0.5–0.5)
1–4 contacts	229,234	(31.6)	297,353	(41.0)	0.8	(0.8–0.8)	0.8	(0.8–0.8)	0.8	(0.8–0.8)
≥ 5 contacts	422,458	(58.2)	243,264	(33.5)	1.7	(1.7–1.7)	1.7	(1.7–1.7)	1.6	(1.6–1.6)
Home visits	162	(0.0)	44	(0.0)	2.1	(1.5–3.0)	2.2	(1.5–3.1)	2.0	(1.4–2.8)
Preventive consultations/services	14,622	(2.0)	14,809	(2.0)	0.8	(0.8–0.8)	0.8	(0.8–0.8)	0.8	(0.8–0.8)
Social-medicine-related	39,165	(5.4)	12,956	(1.8)	2.4	(2.4–2.4)	2.4	(2.4–2.5)	2.3	(2.2–2.3)
GP conversational therapy	4,963	(0.7)	2,733	(0.4)	1.4	(1.4–1.5)	1.4	(1.4–1.5)	1.3	(1.2–1.4)
Reimbursement for chronically ill	1,933	(0.3)	522	(0.1)	2.9	(2.7–3.2)	2.9	(2.6–3.2)	2.3	(2.1–2.5)
Reimbursement for terminally ill	1,164	(0.2)	117	(0.0)	7.9	(6.5–9.5)	7.8	(6.4–9.4)	4.6	(3.8–5.6)
Dementia diagnosis and treatment	363	(0.1)	249	(0.0)	1.2	(1.0–1.4)	1.2	(1.0–1.4)	1.2	(1.0–1.4)
Influenza vaccine	57,447	(7.9)	55,776	(7.7)	0.8	(0.8–0.8)	0.8	(0.8–0.8)	0.8	(0.8–0.8)
Undergoing test at the GP										
Microbiological culture	45,815	(6.3)	23,832	(3.3)	1.5	(1.5–1.6)	1.5	(1.5–1.6)	1.5	(1.5–1.5)
Cytology test	1,543	(0.2)	2,062	(0.3)	0.6	(0.6–0.6)	0.6	(0.6–0.6)	0.6	(0.6–0.7)
Urine test	103,696	(14.3)	64,856	(8.9)	1.3	(1.3–1.3)	1.3	(1.2–1.3)	1.2	(1.2–1.3)
Strep-A test	4,107	(0.6)	2,834	(0.4)	1.2	(1.1–1.2)	1.1	(1.1–1.2)	1.1	(1.1–1.2)
Any blood test	142,898	(19.7)	115,018	(15.8)	1.0	(1.0–1.0)	1.0	(1.0–1.0)	1.0	(1.0–1.0)
C-reactive protein	71,422	(9.8)	31,352	(4.3)	1.8	(1.8–1.8)	1.8	(1.8–1.8)	1.7	(1.7–1.8)
Creatinine	3,233	(0.5)	2,352	(0.3)	1.1	(1.0–1.2)	1.1	(1.0–1.2)	1.1	(1.1–1.2)
Hemoglobin	88,739	(12.2)	57,061	(7.9)	1.2	(1.2–1.2)	1.2	(1.2–1.2)	1.2	(1.2–1.2)
Glucose	66,578	(9.2)	56,367	(7.8)	0.9	(0.9–1.0)	0.9	(0.9–0.9)	0.9	(0.9–0.9)
INR	23,393	(3.2)	17,739	(2.4)	1.0	(1.0–1.1)	1.0	(1.0–1.1)	1.0	(1.0–1.0)

*Sex- and age-adjusted PR, †Sex-, age-, and CCI-adjusted PR

*PR* Prevalence ratio, *CI* Confidence interval, *CCI* Charlson Comorbidity Index, *ES* Doctor from the emergency service, *GP* General practitioner, *INR* International normalized ratio

**Table 5:** Healthcare utilization 1 month prior to preventable hospitalizations

	Cases		Controls		Unadjusted PR		Adjusted PR*		Adjusted PR†	
	*n*	(%)	*n*	(%)	PR	(95% CI)	PR	(95% CI)	PR	(95% CI)
ES consultation or home visit	334,815	(46.1)	213,614	(29.4)	0.9	(0.9–0.9)	0.9	(0.9–0.9)	0.9	(0.9–0.9)
GP consultation or home visit	377,627	(52.0)	227,947	(31.4)	1.0	(1.0–1.0)	1.0	(1.0–1.0 )	1.0	(1.0–1.0)
No contacts	182,167	(25.1)	378,780	(52.2)	0.5	(0.5–0.5)	0.5	(0.5–0.5)	0.5	(0.5–0.5)
1–4 contacts	345,308	(47.6)	281,495	(38.8)	1.2	(1.2–1.2)	1.2	(1.2–1.2)	1.2	(1.2–1.2)
≥ 5 contacts	198,464	(27.3)	65,664	(9.1)	3.0	(3.0–3.1)	3.0	(3.0–3.0)	2.8	(2.8–2.8)
Home visits	84	(0.0)	20	(0.0)	2.0	(1.2–3.2)	2.0	(1.2–3.3)	1.9	(1.1–3.1)
Preventive consultations/services	5,537	(0.8)	5,439	(0.8)	0.6	(0.6–0.6)	0.6	(0.6–0.6)	0.6	(0.6–0.6)
Social-medicine-related	23,235	(3.2)	6,090	(0.8)	2.3	(2.2–2.3)	2.3	(2.3–2.4)	2.2	(2.1–2.3)
GP conversational therapy	2,175	(0.3)	1,150	(0.2)	1.1	(1.0–1.2)	1.1	(1.0–1.2)	1.0	(1.0–1.1)
Reimbursement for chronically ill	831	(0.1)	176	(0.0)	2.8	(2.4–3.3)	2.7	(2.3–3.2)	1.1	(1.8–2.5)
Reimbursement for terminally ill	631	(0.1)	58	(0.0)	6.4	(4.9–8.4)	6.3	(4.8–8.2)	4.0	(3.0–5.2)
Dementia diagnosis and treatment	125	(0.0)	79	(0.0)	0.9	(0.7–1.2)	1.0	(0.7–1.3)	1.0	(0.8–1.3)
Influenza vaccine	17,070	(2.4)	17,252	(2.4)	0.6	(0.6–0.6)	0.6	(0.6–0.6)	0.6	(0.6–0.6)
Undergoing test at the GP										
Microbiological culture	23,393	(3.2)	9,506	(1.3)	1.5	(1.4–1.5)	1.5	(1.5–1.5)	1.5	(1.4–1.5)
Cytology test	495	(0.1)	660	(0.1)	0.4	(0.4–0.5)	0.4	(0.4–0.5)	0.5	(0.4–0.5)
Urine test	54,301	(7.5)	26,830	(3.7)	1.2	(1.2–1.2)	1.2	(1.2–1.2)	1.2	(1.2–1.2)
Strep-A test	2,052	(0.3)	956	(0.1)	1.3	(1.2–1.4)	1.2	(1.1–1.3)	1.3	(1.2–1.4)
Any blood test	68,524	(9.4)	46,719	(6.4)	0.9	(0.9–0.9)	0.9	(0.9–0.9)	0.8	(0.8–0.9)
C-reactive protein	43,565	(6.0)	1,921	(1.6)	2.2	(2.1–2.2)	2.1	(2.1–2.2)	2.1	(2.1–2.1)
Creatinine	1,313	(0.2)	828	(0.1)	0.9	(0.9–1.0)	0.9	(0.9–1.0)	1.0	(0.9–1.1)
Hemoglobin	44,547	(6.1)	21,431	(3.0)	1.2	(1.2–1.3)	1.2	(1.2–1.3)	1.2	(1.2–1.2)
Glucose	29,671	(4.1)	21,507	(3.0)	0.8	(0.8–0.8)	0.8	(0.8–0.8)	0.8	(0.8–0.8)
INR	7,853	(2.5)	14,065	(1.9)	0.8	(0.7–0.8)	0.8	(0.7–0.8)	0.7	(0.7–0.7)

*Sex- and age-adjusted PR, †Sex-, age-, and CCI-adjusted PR

*PR* Prevalence ratio, *CI* Confidence interval, *CCI* Charlson Comorbidity Index, *ES* Doctor from the emergency service, *GP* General practitioner, *INR* International normalized ratio

## Discussion

In this nationwide study of 725,939 older adults with potentially avoidable hospitalizations and 725,939 age- and sex-matched controls, we found that preadmission morbidity was much more prevalent among cases compared to the controls. The strongest associations were seen for chronic lung disease, previous alcohol-related disease, chronic kidney disease, psychiatric disease, dementia, heart failure, and previous hospital contact with infections. Modestly strong associations were seen for previous peptic ulcer disease, cerebrovascular disease, diabetes, cancer, nutrition-related diagnoses, connective tissue disease, and osteoporosis. When we further adjusted for overall comorbidity, a twofold or higher PR was still observed for pre-existing psychiatric disease, chronic lung disease, alcohol-related disease, and previous hospital contact with respiratory tract infections and sepsis. Furthermore, the cases had a high and accelerating number of preadmission contacts with a GP in the months prior to their hospitalization, yet a lower likelihood of GP-out-of-hours contacts, preventive GP consultation, and services, influenza vaccines, or cytology tests.

The strengths of our study include a design with prospectively collected data and the inclusion of all Danish patients with potentially avoidable hospitalizations during the study period, which minimized the risk of recall- and selection bias. Furthermore, our method of density sampling reduced bias due to changes in variables over time. Nevertheless, there are limitations to the study. Data on health status were collected by multiple healthcare professionals at different locations, which could potentially affect the validity of the data. Additionally, the registries utilized in this study are missing important prognostic factors known to impact the risk of hospitalization, including functional and mental status, frailty, nutritional status, and other geriatric aspects [29–32]. Furthermore, due to the length of the study period, it is reasonable to assume that changed follow-up regimes after discharge could affect the results. Moreover, to receive an ICD diagnosis, the patients had to have a prior hospital contact. However, we had two measures to capture diseases, i.e. preadmission hospital diagnoses and medication use in the community. Both measures of diseases were found to be associated with potentially avoidable hospitalizations and, therefore, increased the validity of our identified association. Lastly, since our study was conducted in a publicly funded healthcare system, it may be difficult to generalize our results to countries without universal health insurance coverage, such as the USA.

The higher prevalence of preadmission morbidity and medication use among patients with potentially avoidable hospitalizations is in accordance with two previous studies from the USA [18, 20] and one study from Portugal [16]. In contrast, two other studies from USA [17, 19] showed that dementia was associated with a lower risk of potentially avoidable hospitalizations. The latter two studies only included residents from nursing homes and assisted living facilities, and nursing home residents with dementia in the USA may have fulfilled advanced directives regarding less aggressive treatment preferences, with an increased threshold for hospitalization [17]. On contrary, this current study included both residents from assisted living facilities as well as residents living in their own homes. Furthermore, the contradictory findings could be explained by the differences in healthcare systems between the USA and Denmark. In a Danish setting, other predictors of potentially avoidable hospitalizations have been examined. A previous Danish study examined the association between mobility limitation and potentially avoidable hospitalizations, but found no association [33]. Regarding preadmission healthcare utilization and potentially avoidable hospitalizations for older adults, previous studies have tended to focus on the continuity of care [34–36]. Only one previous study from Taiwan [21] has to our knowledge explored the association with the total number of preadmission physician visits, reporting no association in contrast to our findings.

Potentially preventable hospitalizations may not always be preventable. Understanding and examining them allows for a holistic assessment of healthcare utilization patterns and potential areas for improvement and prevention in the overall healthcare system. For example, fractures resulting from falls or accidents can potentially be prevented through fall prevention programs, home modifications, regular health assessments, anti-osteoporotic medication, and education on safety measures [37–40].

Our findings may point to a feasible avenue for lowering healthcare costs, avoiding unwanted patient outcomes, and improving the well-being of older comorbid patients, by identifying which patient groups are frequently admitted with a diagnosis that could potentially have been avoided. These findings are particularly relevant for preventive initiatives. It has been shown that comprehensive patient assessment, implementation of a care plan that addresses all health-related needs, proactive monitoring, coordination and communication with all care providers, and promotion of the patient, relatives, or caregiver in active engagement of their health are associated with a lower risk of admission [41–47]. However, it is a prerequisite for effective prevention that the municipalities and primary care physicians have extensive training and health information technologies such as telemonitoring devices.

Furthermore, we believe our findings are important for future research that uses potentially avoidable hospitalizations as an indicator of accessibility and quality of primary care, as it provides information about factors that should be taken into consideration for adjustment for baseline differences and case mix. Differences in patient compositions may result in observed differences in rates of potentially avoidable hospitalizations, and these differences are essential to include when benchmarking hospitals and healthcare systems.

Previous studies have primarily investigated the costs of treating ACSCs in the hospital setting. However, a study from the USA [48] investigated the costs of treating ACSC in outpatient, emergency department, and in-patient hospital settings. The study found that in-patient ACSC visits were four times as expensive as emergency department ACSC visits. Furthermore, the cost of emergency department ACSC visits were two times as expensive compared to treating the ACSC in an outpatient clinic. It is probably less costly to treat ACSC in the primary sector as well compared to in-patient hospital settings. Future studies are warranted to examine the costs of treating ACSC in the primary sector compared to hospitals and if it is cost-effective to implement further preventive strategies in the primary sector to lower the rate of potentially preventable hospitalizations.

In conclusion, this nationwide population-based study showed that preadmission morbidity especially chronic lung, heart, and kidney disease, alcohol-related or psychiatric disease including dementia, and previous infections are strongly associated with potentially avoidable hospitalizations and are, therefore, disease areas with high potential for improvement. Furthermore, the cases had a high and accelerating number of preadmission contacts with a GP in the months prior to their hospitalization, which indicates a potential for prevention. These findings highlight the need for tailored clinical initiatives in the community-based healthcare setting for older adults with chronic diseases.

### Supplementary Information

Below is the link to the electronic supplementary material.

Supplementary file1 (PDF 392 KB)

Supplementary file2 (PDF 785 KB)

## Data Availability

As part of the Data Use Agreement at the Danish Data Protection Agency, authors are not allowed to provide raw data.
