# Supplementary material for: Preadmission morbidity and healthcare utilization among older adults with potentially avoidable hospitalizations: a Danish case–control study
Source: Eur Geriatr Med. 2023 Nov 28;15(1):127–38. doi: 10.1007/s41999-023-00887-7 (PMC10876768; doi:10.1007/s41999-023-00887-7)
Supplement: Supplementary file 1 — Supplementary file1 (PDF 392 KB) [file 41999_2023_887_MOESM1_ESM.pdf]

Supplementary Figure 1 Diagnoses and Medication Use Prior to Preventable Hospitalizations

	Sex and age adjusted PR	Sex, age, and CCI adjusted PR
Psychiatric disease:		
D. of psychiatric disease*	2.23(2.15,2.32)	2.07(2.00,2.15)
Any nervous system drugs	1.35(1.35,1.36)	1.29(1.29,1.30)
Psycholeptic drugs	1.41(1.40,1.42)	1.35(1.35,1.36)
Antipsychotics	2.01(1.98,2.03)	1.96(1.93,1.98)
Anxiolytics	1.53(1.52,1.54)	1.47(1.46,1.48)
Hypnotics and sedatives	1.40(1.39,1.41)	1.32(1.31,1.33)
Benzodiazepine derivatives	1.53(1.52,1.54)	1.47(1.46,1.48)
Antidepressant drugs	1.87(1.86,1.89)	1.73(1.72,1.74)
Dementia:		
D. of dementia	2.14(2.09 , 2.19)	1.68(1.64 , 1.72)
Anti-dementia drugs	1.85(1.81 , 1.90)	1.71(1.67 , 1.75)
Cancer:		
D. of cancer	1.73(1.72,1.75)	0.98(0.98,0.99)
Cardiovascular disease:		
Any cardiovascular diagnoses	1.40(1.40,1.41)	1.18(1.17,1.18)
Ischemic heart disease	1.41(1.40,1.42)	1.23(1.22,1.24)
Heart failure	2.22(2.18,2.25)	1.48(1.45,1.51)
Heart valve disease	1.45(1.42,1.48)	1.27(1.24,1.29)
Atrial fibrilation	1.52(1.50,1.53)	1.35(1.33,1.37)
Peripheral artery disease	1.82(1.80,1.85)	1.15(1.13,1.16)
Deep vein thrombosis	1.55(1.51,1.60)	1.37(1.33,1.41)
Hypertension	1.33(1.32,1.35)	1.15(1.13,1.16)
Any cardiovascular medications	1.12(1.12,1.12)	1.04(1.04,1.04)
Antihypertensives except loop-diuretics	1.10(1.10,1.10)	1.05(1.05,1.05)
Agents acting on RAS	1.09(1.08,1.09)	1.00(0.99,1.00)
Other antihypertensives	1.11(1.11,1.12)	1.04(1.04,1.05)
Loop diuretics	1.79(1.78,1.80)	1.59(1.58,1.61)
Digoxin	1.52(1.51,1.54)	1.38(1.36,1.39)
Nitrates	1.41(1.39,1.42)	1.28(1.27,1.30)
Lipid modifying agents	1.09(1.08,1.09)	0.95(0.94,0.95)
NOAC†	1.66(1.62,1.70)	1.43(1.40,1.47)
Any thrombocyte-aggregation prophylaxis	1.24(1.24,1.25)	1.12(1.12,1.13)
Vitamin K antagonists	1.48(1.46,1.50)	1.29(1.27,1.31)
Cerebrovascular disease:		
Any cerebrovascular diagnoses	1.81(1.78,1.83)	1.27(1.25,1.28)
D. of stroke and TIA	1.65(1.63,1.67)	1.16(1.15,1.17)
Diabetes mellitus:		
D. of diabetes	1.78(1.76,1.81)	1.01(1.00,1.03)
Antidiabetics	1.42(1.40,1.43)	1.09(1.08,1.10)
Osteoporosis:		
D. of osteoporosis	1.66(1.63,1.69)	1.55(1.52,1.57)
Chronic lung disease:		
D. of chronic lung disease	3.75(3.69,3.82)	2.59(2.54,2.63)
Asthma/COPD medication	2.45(2.43,2.47)	2.29(2.27,2.31)
Oral steroids	2.43(2.40,2.45)	2.13(2.11,2.16)
Alcoholism-related disease:		
D. of alcoholism-related disease	3.11(3.01,3.22)	2.62(2.53,2.71)
Nutrition-related disease:		
D. of obesity	1.72(1.61,1.83)	1.44(1.34,1.53)
D. of undernutrition	1.82(1.73,1.92)	1.51(1.43,1.60)
Hypertrophia prostatae:		
D. of hypertrophia prostatae	1.21(1.19,1.23)	1.13(1.11,1.14)
Connective tissue disease:		
D. of connective tissue disease	1.61(1.58,1.64)	1.11(1.09,1.13)
Peptic ulcer disease:		
D. of peptic ulcer	1.83(1.79,1.86)	1.20(1.18,1.23)
Gastric acid related drugs	1.59(1.58,1.60)	1.42(1.41,1.43)
Low vision or blindness:		
D. of low vision or blindness	1.39(1.27,1.51)	1.17(1.07,1.27)
Hypercholesterolemia:		
D. of hypercholesterolemia	1.10(1.05,1.16)	0.94(0.90,0.99)
Chronic kidney disease:		
D. of chronic kidney disease	2.42(2.36,2.48)	1.19(1.15,1.22)
Anti-infective:		
Any antibiotic	1.47(1.46,1.47)	1.39(1.39,1.40)
Any D. of infections	2.24(2.22,2.27)	1.89(1.87,1.91)
Antibiotic systemic	1.59(1.58,1.59)	1.50(1.49,1.50)
D. of lower respiratory tract infections	2.86(2.80,2.91)	2.41(2.36,2.46)
D. of skin infections	1.68(1.65,1.71)	1.44(1.41,1.46)
Antibiotic topikal	1.27(1.25,1.29)	1.19(1.17,1.20)
D. of sepsis	3.07(2.99,3.16)	2.39(2.32,2.46)
D. of intestinal infectious diseases	1.62(1.58,1.66)	1.47(1.43,1.51)
Antibiotic Intestinale	3.08(2.98,3.19)	2.57(2.48,2.66)
D. of urinary tract infection	2.30(2.25,2.35)	1.98(1.94,2.02)
Symptoms not classified elsewhere:		
Symptoms not classified elsewhere	1.83(1.81,1.85)	1.59(1.57,1.60)
Observation:		
D. of observation	1.47(1.46,1.47)	1.25(1.24,1.26)

Axis (both panels): 1 2 3 4 5 — Prevalence Ratio with 95% CI

* Diagnosis of psychoses, schizophrenia, affective and personality disorders, †Dabigatran, rivaroxaban, apixaban, and edoxaban
All diagnoses are identified with a lookback period of 10 years from the index date and all medication use is identified with a lookback period of 12 months from the index date
D Diagnosis, PR Prevalence Ratio, CI Confidence Interval, CCI Charlson Comorbidity Index, RAS Renin-Angiotensin System, NOAC Novel Oral Anticoagulants, TIA Transient Ischemic Attack

Supplementary Figure 2 Healthcare Utilization 12, 3, and 1 Month Prior to Preventable Hospitalizations

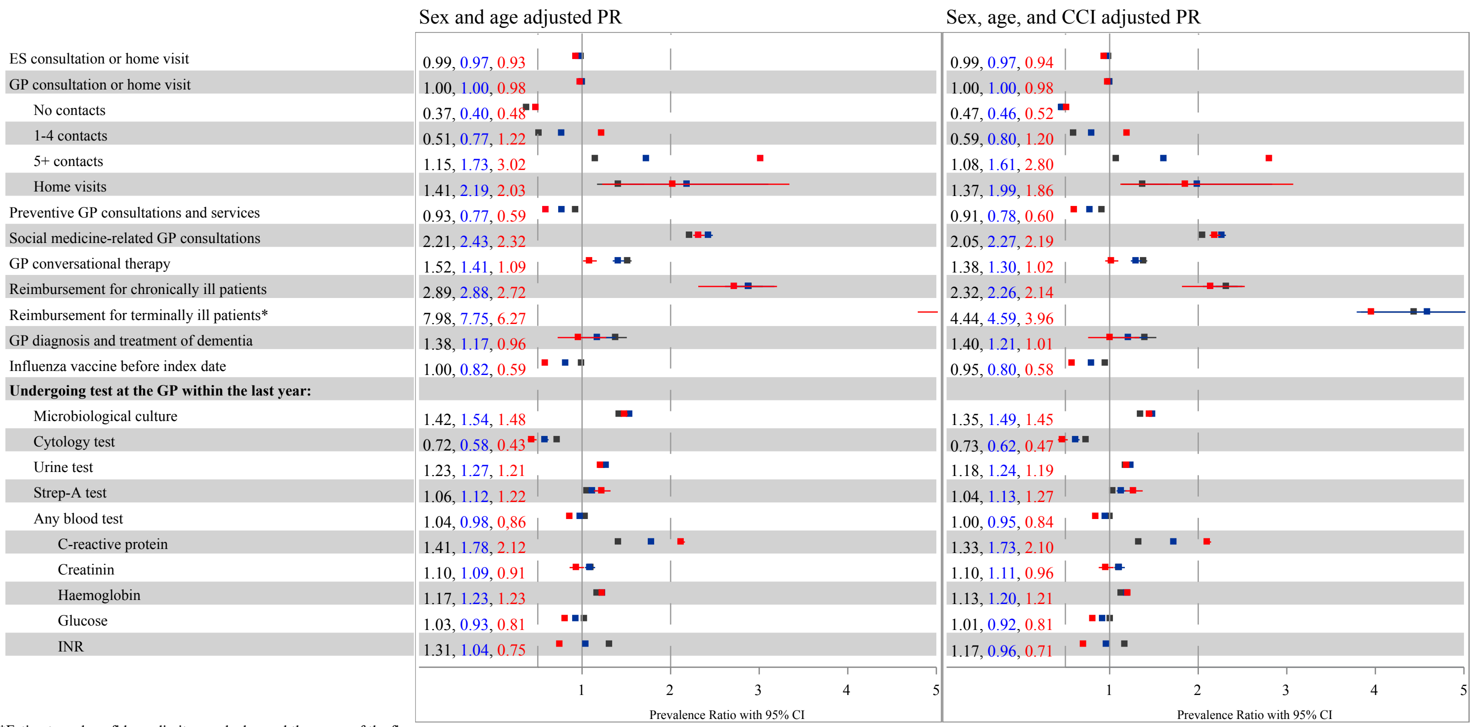

*Estimate and confidence limits may be beyond the scope of the figure
Colour codes: 12-month look back (black), 3-month look back (blue), 1-month look back(red)
PR Prevalence Ratio, CI Confidence Interval, CCI Charlson Comorbidity Index, ES Doctor From the Emergency Service, GP General Practitioner, INR International Normalized Ratio
