# Supplementary material for: Preadmission morbidity and healthcare utilization among older adults with potentially avoidable hospitalizations: a Danish case–control study
Source: Eur Geriatr Med. 2023 Nov 28;15(1):127–38. doi: 10.1007/s41999-023-00887-7 (PMC10876768; doi:10.1007/s41999-023-00887-7)
Supplement: Supplementary file 2 — Supplementary file2 (PDF 785 KB) [file 41999_2023_887_MOESM2_ESM.pdf]

Supplementary Figure 1 Diagnoses and Medication Use Prior to Preventable Hospitalizations Due to Fracture

	Sex and age adjusted PR	Sex, age, and CCI adjusted PR
Psychiatric disease:		
D. of psychiatric disease*	1.83(1.74,1.93)	1.74(1.65,1.82)
Any nervous system drugs	1.20(1.19,1.20)	1.19(1.19,1.19)
Psycholeptic drugs	1.24(1.23,1.25)	1.22(1.21,1.23)
Antipsychotics	1.80(1.76,1.83)	1.76(1.73,1.80)
Anxiolytics	1.29(1.28,1.31)	1.26(1.25,1.28)
Hypnotics and sedatives	1.24(1.23,1.25)	1.21(1.19,1.22)
Benzodiazepine derivatives	1.29(1.28,1.31)	1.26(1.25,1.28)
Antidepressant drugs	1.73(1.71,1.75)	1.65(1.63,1.67)
Dementia:		
D. of dementia	1.99(1.93 , 2.06)	1.75(1.69 , 1.80)
Anti-dementia drugs	1.92(1.85 , 1.98)	1.83(1.77 , 1.89)
Cancer:		
D. of cancer	1.27(1.25,1.29)	0.95(0.94,0.96)
Cardiovascular disease:		
Any cardiovascular diagnoses	1.17(1.16,1.18)	1.06(1.06,1.07)
Ischemic heart disease	1.07(1.05,1.08)	0.98(0.97,1.00)
Heart failure	1.35(1.32,1.39)	1.09(1.06,1.12)
Heart valve disease	1.16(1.13,1.20)	1.08(1.04,1.11)
Atrial fibrilation	1.14(1.12,1.16)	1.06(1.04,1.08)
Peripheral artery disease	1.39(1.36,1.42)	1.08(1.05,1.10)
Deep vein thrombosis	1.23(1.17,1.28)	1.14(1.09,1.19)
Hypertension	1.09(1.07,1.12)	1.01(0.99,1.03)
Any cardiovascular medications	1.02(1.02,1.02)	1.00(1.00,1.00)
Antihypertensives except loop-diuretics	1.00(0.99,1.00)	0.98(0.97,0.98)
Agents acting on RAS	0.96(0.95,0.96)	0.92(0.91,0.93)
Other antihypertensives	0.99(0.99,1.00)	0.96(0.96,0.97)
Loop diuretics	1.27(1.26,1.28)	1.20(1.19,1.22)
Digoxin	1.00(0.98,1.01)	0.95(0.93,0.96)
Nitrates	1.06(1.04,1.08)	1.01(1.00,1.03)
Lipid modifying agents	0.97(0.96,0.98)	0.90(0.89,0.91)
NOAC†	1.22(1.17,1.27)	1.12(1.07,1.16)
Any thrombocyte-aggregation prophylaxis	1.10(1.09,1.11)	1.05(1.04,1.05)
Vitamin K antagonists	1.13(1.10,1.15)	1.04(1.02,1.06)
Cerebrovascular disease:		
Any cerebrovascular diagnoses	1.51(1.48,1.54)	1.24(1.22,1.26)
D. of stroke and TIA	1.41(1.39,1.44)	1.17(1.15,1.18)
Diabetes mellitus:		
D. of diabetes	1.43(1.39,1.46)	1.05(1.03,1.08)
Antidiabetics	1.14(1.12,1.16)	0.99(0.98,1.01)
Osteoporosis:		
D. of osteoporosis	1.67(1.63,1.71)	1.59(1.56,1.63)
Chronic lung disease:		
D. of chronic lung disease	1.52(1.49,1.55)	1.20(1.17,1.22)
Asthma/COPD medication	1.24(1.22,1.25)	1.14(1.13,1.16)
Oral steroids	1.36(1.34,1.38)	1.24(1.22,1.26)
Alcoholism-related disease:		
D. of alcoholism-related disease	3.51(3.33,3.69)	3.10(2.94,3.27)
Nutrition-related disease:		
D. of obesity	1.13(1.02,1.25)	1.02(0.92,1.13)
D. of undernutrition	1.42(1.31,1.54)	1.31(1.21,1.42)
Hypertrophia prostatae:		
D. of hypertrophia prostatae	1.09(1.06,1.13)	1.04(1.01,1.07)
Connective tissue disease:		
D. of connective tissue disease	1.35(1.31,1.39)	1.10(1.07,1.13)
Peptic ulcer disease:		
D. of peptic ulcer	1.53(1.49,1.58)	1.23(1.19,1.26)
Gastric acid related drugs	1.28(1.27,1.30)	1.21(1.20,1.22)
Low vision or blindness:		
D. of low vision or blindness	1.40(1.24,1.58)	1.30(1.15,1.47)
Hypercholesterolemia:		
D. of hypercholesterolemia	0.91(0.84,0.98)	0.84(0.78,0.91)
Chronic kidney disease:		
D. of chronic kidney disease	1.58(1.52,1.65)	1.07(1.03,1.11)
Anti-infective:		
Any antibiotic	1.13(1.12,1.13)	1.09(1.09,1.10)
Any D. of infections	1.42(1.40,1.43)	1.29(1.28,1.31)
Antibiotic systemic	1.15(1.15,1.16)	1.11(1.11,1.12)
D. of lower respiratory tract infections	1.50(1.47,1.53)	1.36(1.34,1.38)
D. of skin infections	1.33(1.29,1.37)	1.22(1.18,1.26)
Antibiotic topikal	1.11(1.09,1.14)	1.08(1.05,1.10)
D. of sepsis	1.57(1.51,1.64)	1.37(1.32,1.43)
D. of intestinal infectious diseases	1.34(1.30,1.38)	1.25(1.21,1.29)
Antibiotic Intestinale	1.55(1.48,1.63)	1.39(1.32,1.46)
D. of urinary tract infection	1.42(1.39,1.46)	1.31(1.28,1.34)
Symptoms not classified elsewhere:		
Symptoms not classified elsewhere	1.47(1.45,1.49)	1.36(1.34,1.38)
Observation:		
D. of observation	1.23(1.23,1.24)	1.13(1.12,1.13)

Axis (both panels): 1 2 3 4 5 — Prevalence Ratio with 95% CI

* Diagnosis of psychoses, schizophrenia, affective and personality disorders, †Dabigatran, rivaroxaban, apixaban, and edoxaban

All diagnoses are identified with a lookback period of 10 years from the index date and all medication use is identified with a lookback period of 12 months from the index date

D Diagnosis, PR Prevalence Ratio, CI Confidence Interval, CCI Charlson Comorbidity Index, RAS Renin Angiotensin System, NOAC Novel Oral Anticoagulants, TIA Transient Ischemic Attack

Cases n = 308,127

Supplementary Figure 2 Healthcare Utilization 12 Months Prior to Preventable Hospitalizations Due to Fracture

	Sex and age adjusted PR	Sex, age, and CCI adjusted PR
ES consultation or home visit	0.98(0.98,0.98)	0.98(0.98,0.98)
GP consultation or home visit	1.00(1.00,1.00)	1.00(1.00,1.00)
No contacts	0.62(0.60,0.63)	0.70(0.68,0.71)
1-4 contacts	0.74(0.73,0.75)	0.80(0.79,0.81)
5+ contacts	1.07(1.07,1.07)	1.04(1.03,1.04)
Home visits	1.28(0.99,1.66)	1.29(1.00,1.68)
Preventive GP consultations and services	0.90(0.89,0.92)	0.90(0.88,0.92)
Social medicine-related GP consultations	1.80(1.77,1.84)	1.73(1.70,1.76)
GP conversational therapy	1.32(1.27,1.38)	1.28(1.22,1.33)
Reimbursement for chronically ill patients	1.84(1.71,1.99)	1.64(1.52,1.78)
Reimbursement for terminally ill patients	3.08(2.48,3.83)	2.33(1.87,2.90)
GP diagnosis and treatment of dementia	1.55(1.36,1.75)	1.55(1.36,1.75)
Influenza vaccine before index date	0.96(0.95,0.97)	0.94(0.93,0.95)
**Undergoing test at the GP within the last year:**		
Microbiological culture	1.19(1.18,1.21)	1.16(1.15,1.18)
Cytology test	0.78(0.74,0.81)	0.78(0.75,0.82)
Urine test	1.09(1.09,1.10)	1.07(1.07,1.08)
Strep-A test	0.85(0.81,0.88)	0.84(0.80,0.87)
Any blood test	0.98(0.97,0.98)	0.96(0.96,0.97)
C-reactive protein	1.03(1.01,1.04)	0.99(0.98,1.00)
Creatinin	0.97(0.92,1.02)	0.97(0.92,1.02)
Haemoglobin	1.03(1.02,1.04)	1.02(1.01,1.03)
Glucose	0.97(0.96,0.98)	0.96(0.95,0.97)
INR	1.08(1.05,1.11)	1.01(0.98,1.04)

Axis: 1 2 3 4 5 — Prevalence Ratio with 95% CI

PR Prevalence Ratio, CI Confidence Interval, CCI Charlson Comorbidity Index, ES Doctor From the Emergency Service, GP General Practitioner, INR International Normalized Ratio

Cases n = 308,127

Supplementary Figure 3 Diagnoses and Medication Use Prior to Preventable Hospitalizations Due to Respiratory Tract Infection

Cases n = 308,127	Sex and age adjusted PR	Sex, age, and CCI adjusted PR
Psychiatric disease:		
D. of psychiatric disease*	2.14(2.04,2.25)	1.93(1.84,2.04)
Any nervous system drugs	1.29(1.28,1.29)	1.32(1.31,1.32)
Psycholeptic drugs	1.53(1.52,1.54)	1.46(1.45,1.47)
Antipsychotics	2.07(2.03,2.11)	2.02(1.98,2.06)
Anxiolytics	1.74(1.72,1.76)	1.67(1.65,1.69)
Hypnotics and sedatives	1.51(1.49,1.52)	1.40(1.39,1.42)
Benzodiazepine derivatives	1.74(1.72,1.76)	1.67(1.65,1.69)
Antidepressant drugs	1.84(1.82,1.86)	1.66(1.64,1.68)
Dementia:		
D. of dementia	1.74(1.68 , 1.79)	1.30(1.26 , 1.35)
Anti-dementia drugs	1.40(1.35 , 1.44)	1.26(1.22 , 1.31)
Cancer:		
D. of cancer	1.74(1.72,1.76)	0.93(0.92,0.94)
Cardiovascular disease:		
Any cardiovascular diagnoses	1.49(1.48,1.49)	1.26(1.25,1.27)
Ischemic heart disease	1.55(1.53,1.57)	1.33(1.31,1.35)
Heart failure	2.65(2.59,2.71)	1.69(1.66,1.73)
Heart valve disease	1.62(1.57,1.66)	1.39(1.35,1.43)
Atrial fibrilation	1.70(1.67,1.73)	1.49(1.47,1.52)
Peripheral artery disease	2.05(2.01,2.09)	1.24(1.21,1.26)
Deep vein thrombosis	1.70(1.63,1.76)	1.47(1.42,1.53)
Hypertension	1.38(1.35,1.41)	1.15(1.12,1.17)
Any cardiovascular medications	1.14(1.14,1.14)	1.05(1.04,1.05)
Antihypertensives except loop-diuretics	1.13(1.13,1.14)	1.07(1.07,1.08)
Agents acting on RAS	1.09(1.08,1.10)	0.98(0.98,0.99)
Other antihypertensives	1.16(1.15,1.17)	1.07(1.07,1.08)
Loop diuretics	2.18(2.16,2.20)	1.95(1.93,1.98)
Digoxin	1.94(1.91,1.97)	1.74(1.71,1.76)
Nitrates	1.67(1.64,1.69)	1.50(1.48,1.53)
Lipid modifying agents	1.09(1.08,1.10)	0.93(0.92,0.94)
NOAC†	1.72(1.67,1.78)	1.47(1.43,1.52)
Any thrombocyte-aggregation prophylaxis	1.28(1.27,1.29)	1.14(1.13,1.15)
Vitamin K antagonists	1.61(1.59,1.64)	1.40(1.38,1.43)
Cerebrovascular disease:		
Any cerebrovascular diagnoses	1.78(1.75,1.81)	1.20(1.19,1.22)
D. of stroke and TIA	1.64(1.61,1.66)	1.11(1.10,1.13)
Diabetes mellitus:		
D. of diabetes	1.73(1.70,1.77)	0.92(0.90,0.94)
Antidiabetics	1.40(1.38,1.42)	1.04(1.02,1.05)
Osteoporosis:		
D. of osteoporosis	1.72(1.68,1.75)	1.55(1.52,1.58)
Chronic lung disease:		
D. of chronic lung disease	6.26(6.12,6.40)	4.47(4.37,4.58)
Asthma/COPD medication	4.04(3.99,4.08)	3.93(3.88,3.97)
Oral steroids	3.58(3.53,3.63)	3.20(3.16,3.25)
Alcoholism-related disease:		
D. of alcoholism-related disease	2.73(2.61,2.85)	2.23(2.13,2.33)
Nutrition-related disease:		
D. of obesity	1.98(1.81,2.16)	1.56(1.43,1.71)
D. of undernutrition	2.25(2.08,2.43)	1.75(1.62,1.90)
Hypertrophia prostatae:		
D. of hypertrophia prostatae	1.12(1.10,1.14)	1.04(1.02,1.06)
Connective tissue disease:		
D. of connective tissue disease	1.78(1.73,1.83)	1.16(1.13,1.19)
Peptic ulcer disease:		
D. of peptic ulcer	1.90(1.86,1.95)	1.19(1.16,1.22)
Gastric acid related drugs	1.66(1.65,1.67)	1.45(1.44,1.46)
Low vision or blindness:		
D. of low vision or blindness	1.34(1.17,1.53)	1.09(0.95,1.25)
Hypercholesterolemia:		
D. of hypercholesterolemia	1.10(1.03,1.18)	0.90(0.84,0.97)
Chronic kidney disease:		
D. of chronic kidney disease	2.46(2.38,2.54)	1.16(1.12,1.19)
Anti-infective:		
Any antibiotic	1.72(1.71,1.73)	1.64(1.64,1.65)
Any D. of infections	2.67(2.63,2.71)	2.25(2.21,2.28)
Antibiotic systemic	1.91(1.90,1.92)	1.82(1.81,1.83)
D. of lower respiratory tract infections	4.40(4.29,4.51)	3.76(3.67,3.86)
D. of skin infections	1.84(1.80,1.89)	1.53(1.50,1.58)
Antibiotic topikal	1.34(1.31,1.36)	1.23(1.20,1.25)
D. of sepsis	3.11(3.01,3.21)	2.41(2.33,2.50)
D. of intestinal infectious diseases	1.70(1.65,1.74)	1.49(1.45,1.53)
Antibiotic Intestinale	3.90(3.73,4.08)	3.25(3.11,3.40)
D. of urinary tract infection	2.11(2.07,2.16)	1.79(1.75,1.83)
Symptoms not classified elsewhere:		
Symptoms not classified elsewhere	1.85(1.83,1.88)	1.57(1.55,1.59)
Observation:		
D. of observation	1.50(1.49,1.51)	1.27(1.26,1.28)

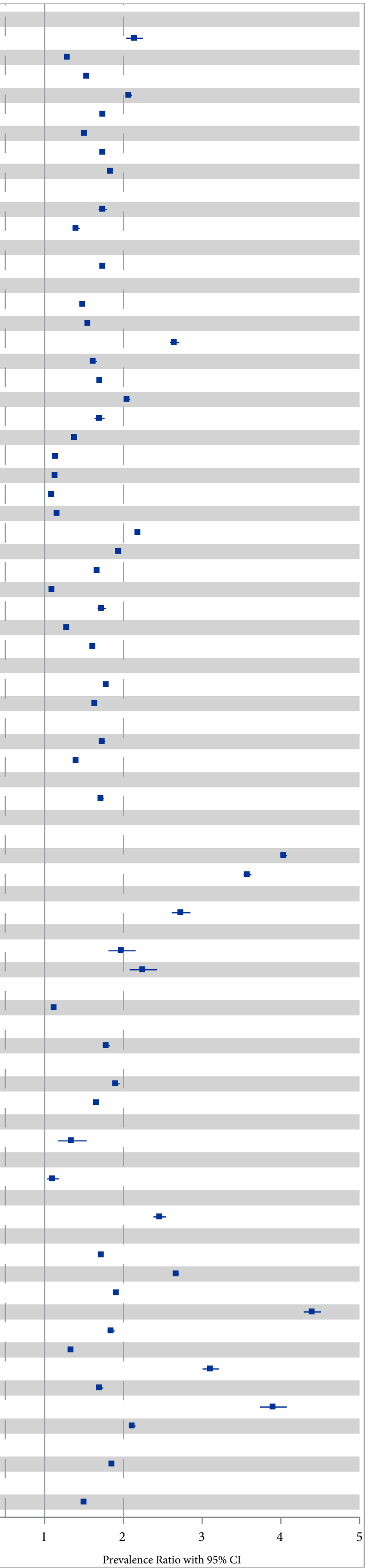

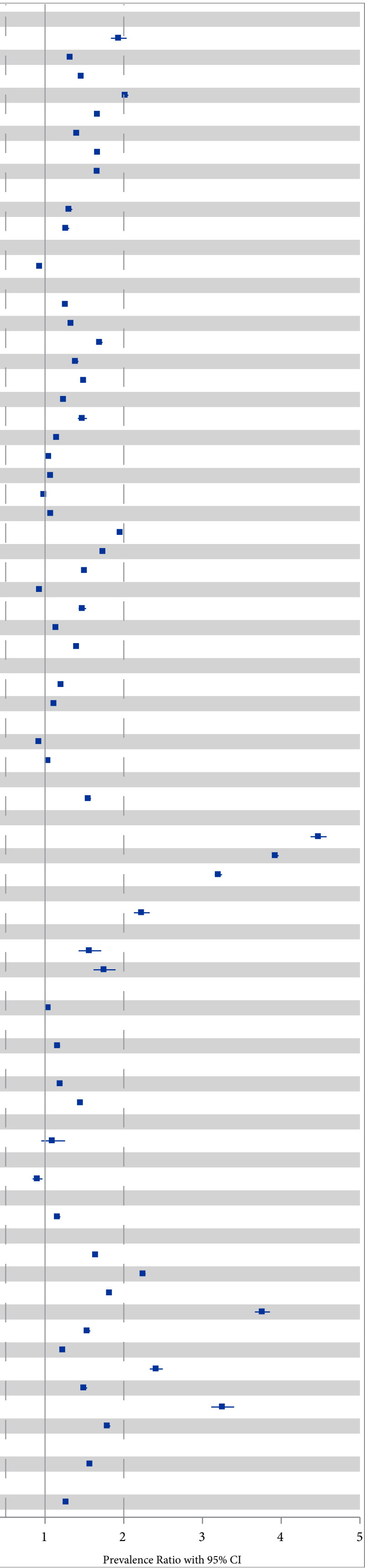

* Diagnosis of psychoses, schizophrenia, affective and personality disorders, †Dabigatran, rivaroxaban, apixaban, and edoxaban
All diagnoses are identified with a lookback period of 10 years from the index date and all medication use is identified with a lookback period of 12 months from the index date
D Diagnosis, PR Prevalence Ratio, CI Confidence Interval, CCI Charlson Comorbidity Index, RAS Renin Angiotensin System, NOAC Novel Oral Anticoagulants, TIA Transient Ischemic Attack

Cases n = 356,231

Supplementary Figure 4 Healthcare Utilization 12 Months Prior to Preventable Hospitalizations Due to Respiratory Tract Infection

	Sex and age adjusted PR	Sex, age, and CCI adjusted PR
ES consultation or home visit	0.99(0.99,0.99)	0.99(0.99,0.99)
GP consultation or home visit	1.01(1.01,1.01)	1.01(1.00,1.01)
No contacts	0.21(0.21,0.22)	0.29(0.28,0.29)
1-4 contacts	0.37(0.37,0.38)	0.45(0.44,0.45)
5+ contacts	1.17(1.16,1.17)	1.09(1.09,1.10)
Home visits	1.40(1.04,1.89)	1.38(1.01,1.86)
Preventive GP consultations and services	0.93(0.92,0.95)	0.92(0.90,0.94)
Social medicine-related GP consultations	2.14(2.10,2.18)	1.95(1.92,1.99)
GP conversational therapy	1.42(1.36,1.48)	1.27(1.22,1.32)
Reimbursement for chronically ill patients	3.09(2.89,3.30)	2.46(2.30,2.64)
Reimbursement for terminally ill patients	6.83(5.63,8.28)	3.81(3.14,4.63)
GP diagnosis and treatment of dementia	1.11(0.98,1.27)	1.17(1.02,1.34)
Influenza vaccine before index date	1.03(1.02,1.04)	0.98(0.97,0.99)
**Undergoing test at the GP within the last year:**		
Microbiological culture	1.34(1.33,1.36)	1.26(1.24,1.28)
Cytology test	0.65(0.62,0.68)	0.67(0.63,0.70)
Urine test	1.17(1.16,1.18)	1.12(1.11,1.13)
Strep-A test	1.21(1.17,1.25)	1.18(1.14,1.23)
Any blood test	1.04(1.03,1.04)	1.00(1.00,1.01)
C-reactive protein	1.68(1.67,1.70)	1.59(1.57,1.61)
Creatinin	1.10(1.05,1.15)	1.11(1.06,1.16)
Haemoglobin	1.21(1.20,1.22)	1.16(1.15,1.17)
Glucose	1.01(1.00,1.02)	1.00(0.99,1.01)
INR	1.41(1.38,1.44)	1.26(1.23,1.29)

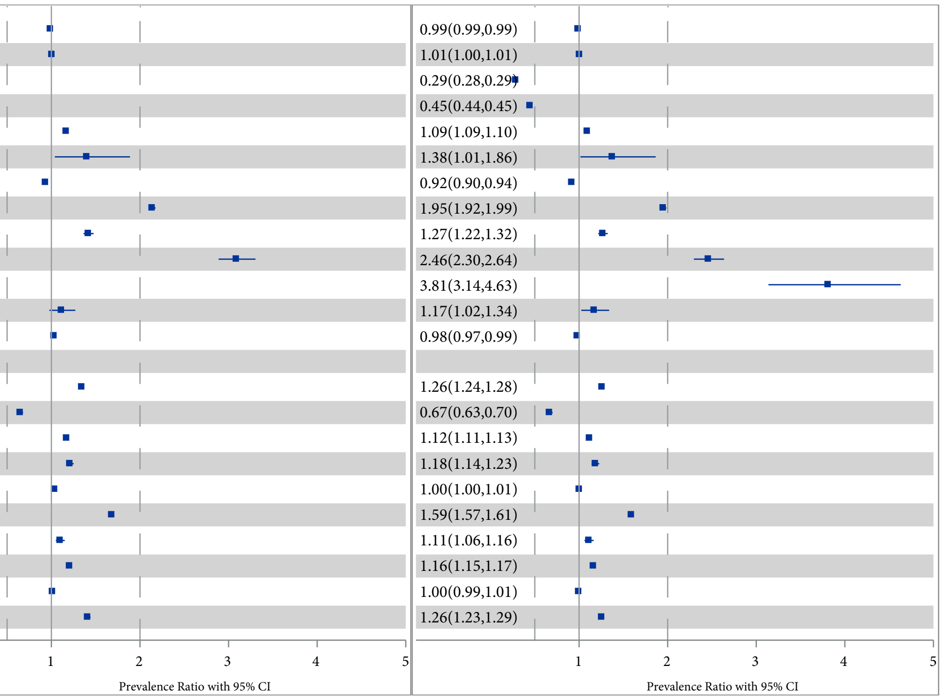

PR Prevalence Ratio, CI Confidence Interval, CCI Charlson Comorbidity Index, ES Doctor From the Emergency Service, GP General Practitioner, INR International Normalized Ratio

Cases n = 356,231

Supplementary Figure 5 Diagnoses and Medication Use Prior to Preventable Hospitalizations Due to Dehydration

	Sex and age adjusted PR	Sex, age, and CCI adjusted PR
Psychiatric disease:		
D. of psychiatric disease*	2.80(2.59,3.02)	2.67(2.46,2.89)
Any nervous system drugs	1.38(1.37,1.39)	1.32(1.31,1.33)
Psycholeptic drugs	1.50(1.48,1.52)	1.44(1.42,1.45)
Antipsychotics	2.37(2.30,2.45)	2.41(2.33,2.49)
Anxiolytics	1.67(1.63,1.70)	1.60(1.57,1.64)
Hypnotics and sedatives	1.45(1.43,1.48)	1.35(1.33,1.37)
Benzodiazepine derivatives	1.66(1.63,1.70)	1.60(1.56,1.64)
Antidepressant drugs	1.95(1.91,1.98)	1.80(1.77,1.83)
Dementia:		
D. of dementia	2.50(2.39,2.62)	1.88(1.80,1.97)
Anti-dementia drugs	1.99(1.90,2.08)	1.84(1.76,1.93)
Cancer:		
D. of cancer	2.20(2.15,2.24)	1.03(1.01,1.05)
Cardiovascular disease:		
Any cardiovascular diagnoses	1.46(1.45,1.48)	1.20(1.19,1.21)
Ischemic heart disease	1.46(1.43,1.49)	1.23(1.20,1.26)
Heart failure	2.58(2.49,2.67)	1.61(1.55,1.66)
Heart valve disease	1.34(1.28,1.40)	1.13(1.08,1.18)
Atrial fibrilation	1.61(1.56,1.65)	1.39(1.35,1.43)
Peripheral artery disease	1.83(1.77,1.90)	1.04(1.01,1.07)
Deep vein thrombosis	1.71(1.61,1.82)	1.47(1.38,1.57)
Hypertension	1.57(1.52,1.62)	1.33(1.29,1.37)
Any cardiovascular medications	1.14(1.14,1.15)	1.07(1.06,1.07)
Antihypertensives except loop-diuretics	1.16(1.15,1.17)	1.11(1.10,1.11)
Agents acting on RAS	1.22(1.21,1.24)	1.13(1.11,1.14)
Other antihypertensives	1.17(1.15,1.18)	1.08(1.07,1.10)
Loop diuretics	1.86(1.84,1.89)	1.60(1.58,1.63)
Digoxin	1.87(1.82,1.92)	1.67(1.62,1.71)
Nitrates	1.38(1.34,1.41)	1.22(1.19,1.25)
Lipid modifying agents	1.13(1.11,1.15)	0.94(0.93,0.96)
NOAC†	1.93(1.84,2.03)	1.62(1.54,1.70)
Any thrombocyte-aggregation prophylaxis	1.29(1.27,1.30)	1.16(1.14,1.17)
Vitamin K antagonists	1.40(1.36,1.44)	1.19(1.16,1.23)
Cerebrovascular disease:		
Any cerebrovascular diagnoses	2.03(1.98,2.08)	1.35(1.32,1.39)
D. of stroke and TIA	1.86(1.82,1.91)	1.25(1.22,1.28)
Diabetes mellitus:		
D. of diabetes	2.17(2.10,2.26)	1.06(1.02,1.10)
Antidiabetics	1.59(1.55,1.63)	1.16(1.13,1.19)
Osteoporosis:		
D. of osteoporosis	1.43(1.38,1.48)	1.30(1.25,1.34)
Chronic lung disease:		
D. of chronic lung disease	1.80(1.75,1.86)	1.07(1.04,1.11)
Asthma/COPD medication	1.34(1.32,1.37)	1.09(1.07,1.12)
Oral steroids	1.84(1.80,1.89)	1.46(1.42,1.50)
Alcoholism-related disease:		
D. of alcoholism-related disease	4.22(3.88,4.58)	3.63(3.33,3.97)
Nutrition-related disease:		
D. of obesity	1.76(1.48,2.08)	1.39(1.16,1.67)
D. of undernutrition	3.32(2.88,3.83)	2.53(2.18,2.94)
Hypertrophia prostatae:		
D. of hypertrophia prostatae	1.11(1.07,1.15)	1.03(0.99,1.07)
Connective tissue disease:		
D. of connective tissue disease	1.52(1.45,1.59)	0.94(0.90,0.99)
Peptic ulcer disease:		
D. of peptic ulcer	2.05(1.96,2.14)	1.24(1.19,1.29)
Gastric acid related drugs	1.79(1.76,1.81)	1.55(1.53,1.57)
Low vision or blindness:		
D. of low vision or blindness	1.43(1.09,1.88)	1.18(0.90,1.56)
Hypercholesterolemia:		
D. of hypercholesterolemia	1.02(0.90,1.16)	0.81(0.71,0.92)
Chronic kidney disease:		
D. of chronic kidney disease	2.85(2.70,3.01)	1.25(1.18,1.32)
Anti-infective:		
Any antibiotic	1.43(1.42,1.44)	1.33(1.32,1.34)
Any D. of infections	2.13(2.09,2.17)	1.79(1.75,1.82)
Antibiotic systemic	1.51(1.50,1.53)	1.40(1.38,1.41)
D. of lower respiratory tract infections	2.18(2.13,2.23)	1.81(1.77,1.85)
D. of skin infections	1.81(1.74,1.89)	1.49(1.43,1.56)
Antibiotic topikal	1.28(1.24,1.33)	1.17(1.13,1.21)
D. of sepsis	3.13(2.98,3.30)	2.42(2.29,2.55)
D. of intestinal infectious diseases	2.64(2.52,2.76)	2.28(2.17,2.39)
Antibiotic Intestinale	3.75(3.50,4.01)	3.09(2.88,3.31)
D. of urinary tract infection	2.49(2.41,2.56)	2.15(2.09,2.22)
Symptoms not classified elsewhere:		
Symptoms not classified elsewhere	2.11(2.07,2.16)	1.81(1.77,1.85)
Observation:		
D. of observation	1.52(1.50,1.53)	1.26(1.25,1.27)

Prevalence Ratio with 95% CI (axis 1–5)

Prevalence Ratio with 95% CI (axis 1–5)

* Diagnosis of psychoses, schizophrenia, affective and personality disorders, †Dabigatran, rivaroxaban, apixaban, and edoxaban
All diagnoses are identified with a lookback period of 10 years from the index date and all medication use is identified with a lookback period of 12 months from the index date
D Diagnosis, PR Prevalence Ratio, CI Confidence Interval, CCI Charlson Comorbidity Index, RAS Renin Angiotensin System, NOAC Novel Oral Anticoagulants, TIA Transient Ischemic Attack

Cases n = 104,598

Supplementary Figure 6 Healthcare Utilization 12 Months Prior to Preventable Hospitalizations Due to Dehydration

	Sex and age adjusted PR	Sex, age, and CCI adjusted PR
ES consultation or home visit	0.97(0.97,0.97)	0.96(0.96,0.97)
GP consultation or home visit	1.01(1.01,1.01)	1.01(1.00,1.01)
No contacts	0.26(0.24,0.27)	0.39(0.37,0.42)
1-4 contacts	0.35(0.34,0.36)	0.45(0.43,0.46)
5+ contacts	1.13(1.12,1.13)	1.10(1.09,1.10)
Home visits	1.23(0.77,1.95)	1.23(0.77,1.98)
Preventive GP consultations and services	0.95(0.92,0.98)	0.95(0.92,0.98)
Social medicine-related GP consultations	2.56(2.50,2.63)	2.41(2.34,2.47)
GP conversational therapy	1.66(1.54,1.78)	1.46(1.36,1.57)
Reimbursement for chronically ill patients	2.61(2.35,2.91)	2.02(1.81,2.26)
Reimbursement for terminally ill patients	8.79(6.80,11.38)	4.60(3.54,5.98)
GP diagnosis and treatment of dementia	1.71(1.40,2.10)	1.81(1.47,2.23)
Influenza vaccine before index date	0.91(0.90,0.92)	0.86(0.85,0.87)
**Undergoing test at the GP within the last year:**		
Microbiological culture	1.53(1.50,1.56)	1.45(1.42,1.48)
Cytology test	0.57(0.51,0.63)	0.57(0.51,0.64)
Urine test	1.31(1.29,1.33)	1.26(1.24,1.27)
Strep-A test	0.90(0.84,0.97)	0.86(0.79,0.93)
Any blood test	1.01(1.00,1.02)	0.98(0.97,0.99)
C-reactive protein	1.22(1.20,1.24)	1.12(1.09,1.14)
Creatinin	1.34(1.23,1.46)	1.40(1.28,1.53)
Haemoglobin	1.17(1.15,1.18)	1.11(1.10,1.13)
Glucose	0.99(0.97,1.00)	0.98(0.96,0.99)
INR	1.26(1.22,1.31)	1.09(1.05,1.14)

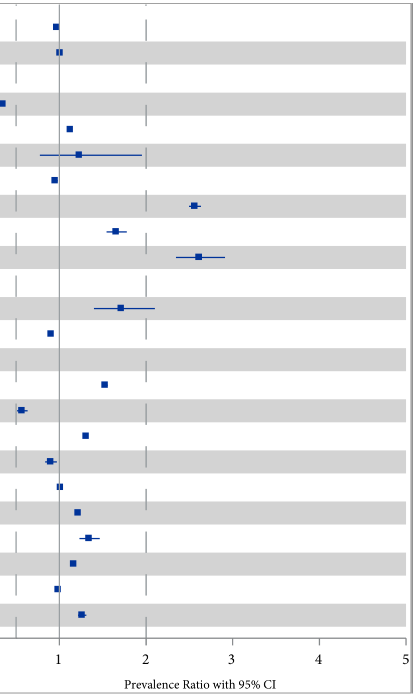

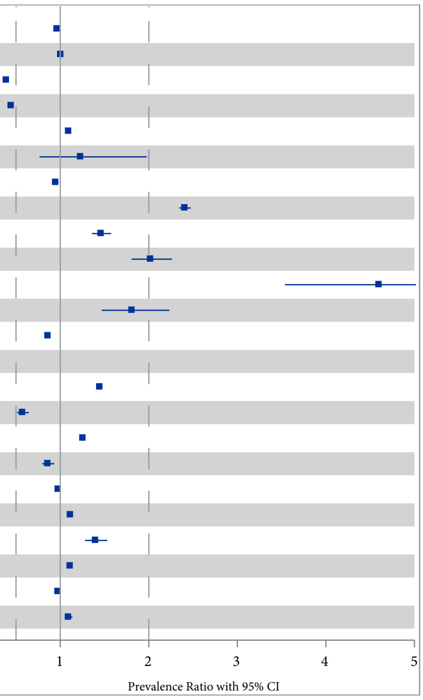

PR Prevalence Ratio, CI Confidence Interval, CCI Charlson Comorbidity Index, ES Doctor From the Emergency Service, GP General Practitioner, INR International Normalized Ratio

Cases n = 104,598

Supplementary Figure 7 Diagnoses and Medication Use Prior to Preventable Hospitalizations Due to UTI

	Sex and age adjusted PR	Sex, age, and CCI adjusted PR
Psychiatric disease:		
D. of psychiatric disease*	2.67(2.44,2.92)	2.44(2.23,2.68)
Any nervous system drugs	1.35(1.34,1.36)	1.16(1.16,1.17)
Psycholeptic drugs	1.44(1.42,1.46)	1.37(1.35,1.39)
Antipsychotics	2.16(2.08,2.24)	2.11(2.03,2.19)
Anxiolytics	1.56(1.53,1.60)	1.50(1.46,1.53)
Hypnotics and sedatives	1.40(1.37,1.42)	1.31(1.28,1.33)
Benzodiazepine derivatives	1.56(1.52,1.60)	1.50(1.46,1.53)
Antidepressant drugs	1.88(1.84,1.91)	1.72(1.68,1.75)
Dementia:		
D. of dementia	2.45(2.32,2.58)	1.86(1.76,1.96)
Anti-dementia drugs	1.87(1.77,1.98)	1.73(1.63,1.83)
Cancer:		
D. of cancer	1.75(1.71,1.79)	0.93(0.91,0.94)
Cardiovascular disease:		
Any cardiovascular diagnoses	1.46(1.44,1.48)	1.21(1.20,1.22)
Ischemic heart disease	1.45(1.41,1.49)	1.24(1.21,1.27)
Heart failure	1.94(1.86,2.03)	1.24(1.19,1.29)
Heart valve disease	1.27(1.21,1.34)	1.09(1.04,1.15)
Atrial fibrillation	1.59(1.54,1.64)	1.38(1.34,1.43)
Peripheral artery disease	1.75(1.68,1.82)	1.04(1.00,1.08)
Deep vein thrombosis	1.66(1.55,1.78)	1.46(1.36,1.56)
Hypertension	1.48(1.42,1.53)	1.26(1.21,1.30)
Any cardiovascular medications	1.13(1.12,1.13)	1.06(1.05,1.06)
Antihypertensives except loop-diuretics	1.11(1.10,1.12)	1.06(1.05,1.06)
Agents acting on RAS	1.08(1.06,1.09)	0.99(0.97,1.00)
Other antihypertensives	1.13(1.12,1.15)	1.06(1.04,1.07)
Loop diuretics	1.64(1.61,1.66)	1.42(1.39,1.45)
Digoxin	1.61(1.56,1.66)	1.42(1.38,1.46)
Nitrates	1.38(1.34,1.42)	1.23(1.19,1.27)
Lipid modifying agents	1.11(1.09,1.13)	0.95(0.93,0.96)
NOAC†	1.84(1.74,1.95)	1.56(1.48,1.66)
Any thrombocyte-aggregation prophylaxis	1.32(1.30,1.33)	1.18(1.17,1.20)
Vitamin K antagonists	1.44(1.39,1.49)	1.24(1.20,1.29)
Cerebrovascular disease:		
Any cerebrovascular diagnoses	2.19(2.13,2.25)	1.52(1.47,1.56)
D. of stroke and TIA	2.01(1.95,2.06)	1.39(1.36,1.43)
Diabetes mellitus:		
D. of diabetes	2.36(2.27,2.46)	1.27(1.22,1.32)
Antidiabetics	1.77(1.72,1.81)	1.31(1.28,1.35)
Osteoporosis:		
D. of osteoporosis	1.44(1.38,1.50)	1.30(1.25,1.35)
Chronic lung disease:		
D. of chronic lung disease	1.81(1.74,1.87)	1.15(1.11,1.19)
Asthma/COPD medication	1.32(1.29,1.35)	1.11(1.08,1.13)
Oral steroids	1.74(1.69,1.79)	1.43(1.39,1.47)
Alcoholism-related disease:		
D. of alcoholism-related disease	2.57(2.34,2.82)	2.04(1.86,2.25)
Nutrition-related disease:		
D. of obesity	2.67(2.23,3.20)	2.13(1.77,2.58)
D. of undernutrition	2.05(1.76,2.40)	1.61(1.38,1.89)
Hypertrophia prostatae:		
D. of hypertrophia prostatae	1.97(1.90,2.05)	1.93(1.86,2.01)
Connective tissue disease:		
D. of connective tissue disease	1.66(1.58,1.75)	1.08(1.02,1.14)
Peptic ulcer disease:		
D. of peptic ulcer	1.83(1.75,1.92)	1.15(1.10,1.21)
Gastric acid related drugs	1.66(1.63,1.68)	1.46(1.44,1.48)
Low vision or blindness:		
D. of low vision or blindness	1.72(1.32,2.24)	1.43(1.09,1.88)
Hypercholesterolemia:		
D. of hypercholesterolemia	1.36(1.19,1.56)	1.11(0.96,1.28)
Chronic kidney disease:		
D. of chronic kidney disease	3.01(2.83,3.20)	1.43(1.35,1.52)
Anti-infective:		
Any antibiotic	1.68(1.67,1.70)	1.59(1.58,1.61)
Any D. of infections	2.36(2.31,2.40)	2.00(1.96,2.04)
Antibiotic systemic	1.87(1.85,1.88)	1.76(1.74,1.78)
D. of lower respiratory tract infections	2.30(2.24,2.36)	1.92(1.87,1.98)
D. of skin infections	1.96(1.87,2.06)	1.66(1.58,1.74)
Antibiotic topikal	1.40(1.35,1.46)	1.30(1.25,1.35)
D. of sepsis	5.35(5.05,5.67)	4.37(4.12,4.64)
D. of intestinal infectious diseases	2.33(2.21,2.46)	2.03(1.93,2.15)
Antibiotic Intestinale	3.06(2.82,3.31)	2.47(2.28,2.68)
D. of urinary tract infection	5.70(5.45,5.95)	5.14(4.91,5.38)
Symptoms not classified elsewhere:		
Symptoms not classified elsewhere	2.10(2.05,2.15)	1.82(1.78,1.87)
Observation:		
D. of observation	1.55(1.54,1.57)	1.33(1.31,1.34)

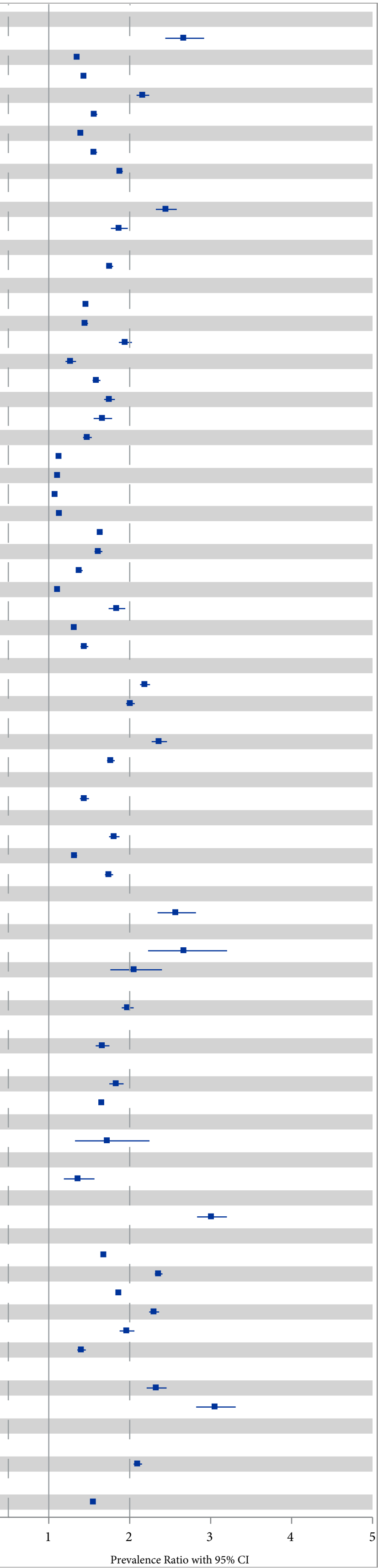

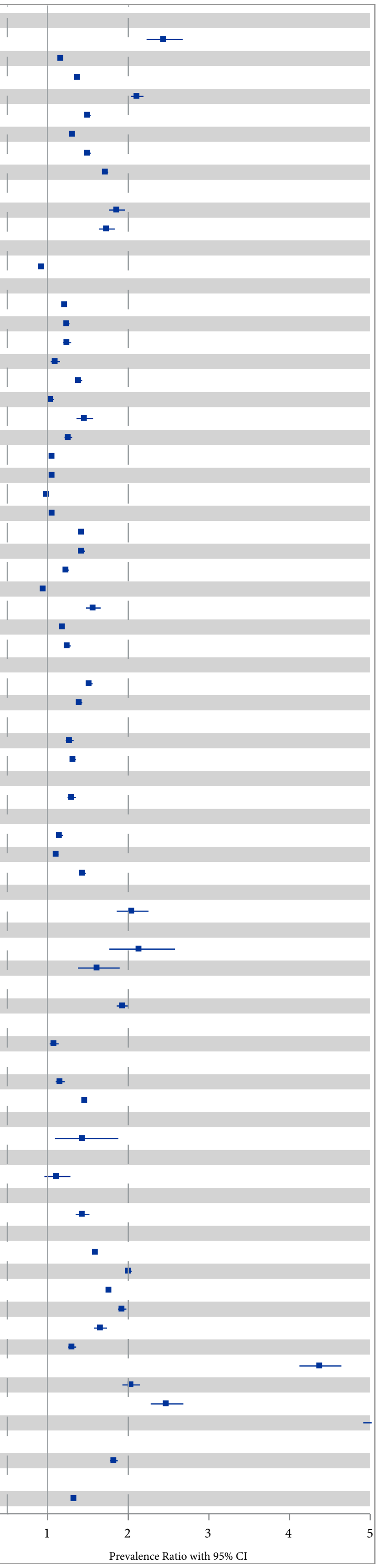

* Diagnosis of psychoses, schizophrenia, affective and personality disorders, †Dabigatran, rivaroxaban, apixaban, and edoxaban
All diagnoses are identified with a lookback period of 10 years from the index date and all medication use is identified with a lookback period of 12 months from the index date
D Diagnosis, PR Prevalence Ratio, CI Confidence Interval, CCI Charlson Comorbidity Index, RAS Renin Angiotensin System, NOAC Novel Oral Anticoagulants, TIA Transient Ischemic Attack

Cases n = 85,330

Supplementary Figure 8 Healthcare Utilization 12 Months Prior to Preventable Hospitalizations Due to UTI

	Sex and age adjusted PR	Sex, age, and CCI adjusted PR
ES consultation or home visit	0.99(0.99,0.99)	0.98(0.98,0.99)
GP consultation or home visit	1.01(1.01,1.01)	1.01(1.00,1.01)
No contacts	0.20(0.19,0.22)	0.28(0.26,0.30)
1-4 contacts	0.32(0.31,0.33)	0.39(0.38,0.41)
5+ contacts	1.15(1.15,1.16)	1.10(1.10,1.11)
Home visits	1.33(0.75,2.37)	1.32(0.73,2.37)
Preventive GP consultations and services	0.91(0.87,0.94)	0.89(0.85,0.92)
Social medicine-related GP consultations	2.38(2.31,2.46)	2.20(2.13,2.27)
GP conversational therapy	1.26(1.16,1.37)	1.14(1.05,1.24)
Reimbursement for chronically ill patients	2.43(2.15,2.75)	1.91(1.69,2.17)
Reimbursement for terminally ill patients	4.59(3.28,6.42)	2.51(1.78,3.53)
GP diagnosis and treatment of dementia	1.55(1.23,1.95)	1.59(1.26,2.02)
Influenza vaccine before index date	0.92(0.91,0.94)	0.88(0.87,0.89)
**Undergoing test at the GP within the last year:**		
Microbiological culture	2.52(2.47,2.58)	2.44(2.39,2.49)
Cytology test	0.86(0.78,0.96)	0.89(0.80,1.00)
Urine test	1.89(1.87,1.92)	1.85(1.82,1.87)
Strep-A test	1.00(0.93,1.08)	0.99(0.91,1.07)
Any blood test	0.99(0.98,1.00)	0.96(0.95,0.97)
C-reactive protein	1.24(1.22,1.27)	1.16(1.13,1.18)
Creatinin	1.02(0.93,1.12)	1.03(0.94,1.14)
Haemoglobin	1.10(1.08,1.12)	1.06(1.04,1.08)
Glucose	1.00(0.98,1.02)	0.98(0.97,1.00)
INR	1.20(1.15,1.25)	1.05(1.01,1.10)

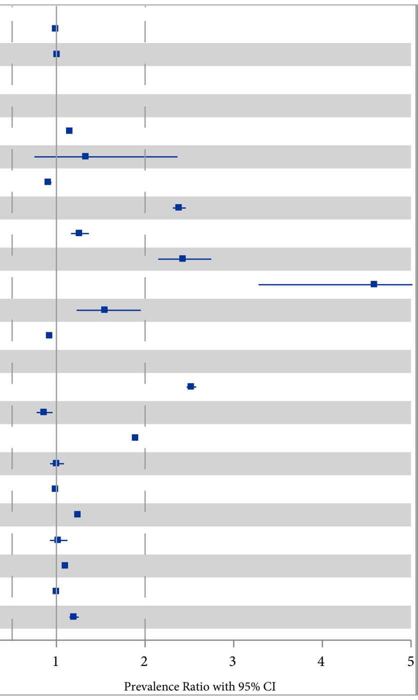

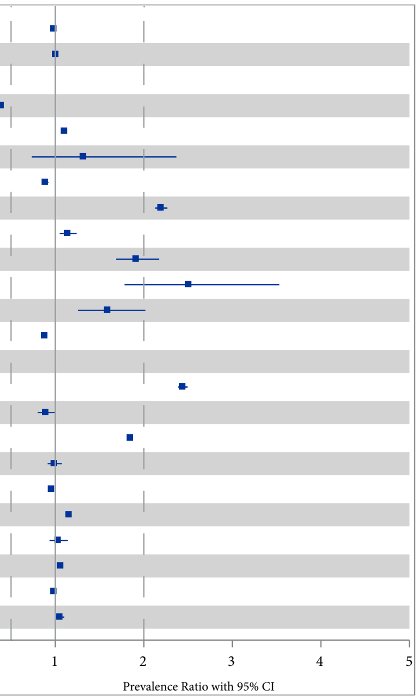

PR Prevalence Ratio, CI Confidence Interval, CCI Charlson Comorbidity Index, ES Doctor From the Emergency Service, GP General Practitioner, INR International Normalized Ratio

Cases n = 85,330

Supplementary Figure 9 Diagnoses and Medication Use Prior to Preventable Hospitalizations Due to Obstipation

	Sex and age adjusted PR	Sex, age, and CCI adjusted PR
Psychiatric disease:		
D. of psychiatric disease*	2.73(2.42,3.09)	2.59(2.28,2.94)
Any nervous system drugs	1.50(1.48,1.51)	1.20(1.19,1.21)
Psycholeptic drugs	1.69(1.66,1.72)	1.61(1.58,1.64)
Antipsychotics	2.10(2.00,2.20)	2.09(1.98,2.20)
Anxiolytics	1.91(1.86,1.97)	1.82(1.76,1.88)
Hypnotics and sedatives	1.78(1.74,1.82)	1.65(1.61,1.69)
Benzodiazepine derivatives	1.92(1.86,1.98)	1.83(1.77,1.89)
Antidepressant drugs	1.89(1.84,1.93)	1.71(1.66,1.75)
Dementia:		
D. of dementia	1.47(1.36 , 1.60)	1.06(0.97 , 1.16)
Anti-dementia drugs	1.13(1.03 , 1.23)	1.01(0.92 , 1.11)
Cancer:		
D. of cancer	2.59(2.52,2.67)	1.16(1.13,1.19)
Cardiovascular disease:		
Any cardiovascular diagnoses	1.49(1.47,1.52)	1.19(1.17,1.21)
Ischemic heart disease	1.66(1.61,1.71)	1.39(1.35,1.44)
Heart failure	2.18(2.06,2.32)	1.27(1.20,1.35)
Heart valve disease	1.30(1.21,1.40)	1.08(1.00,1.16)
Atrial fibrilation	1.49(1.43,1.56)	1.28(1.22,1.34)
Peripheral artery disease	2.03(1.93,2.14)	1.11(1.06,1.17)
Deep vein thrombosis	1.92(1.74,2.10)	1.62(1.47,1.79)
Hypertension	1.54(1.47,1.62)	1.27(1.20,1.33)
Any cardiovascular medications	1.16(1.15,1.17)	1.05(1.04,1.06)
Antihypertensives except loop-diuretics	1.12(1.11,1.14)	1.06(1.05,1.07)
Agents acting on RAS	1.05(1.03,1.07)	0.94(0.92,0.96)
Other antihypertensives	1.12(1.10,1.14)	1.03(1.01,1.05)
Loop diuretics	1.72(1.68,1.77)	1.44(1.40,1.47)
Digoxin	1.52(1.46,1.59)	1.33(1.27,1.39)
Nitrates	1.73(1.66,1.80)	1.53(1.47,1.60)
Lipid modifying agents	1.14(1.11,1.17)	0.95(0.92,0.97)
NOAC†	1.54(1.41,1.67)	1.25(1.14,1.36)
Any thrombocyte-aggregation prophylaxis	1.31(1.29,1.33)	1.15(1.13,1.17)
Vitamin K antagonists	1.28(1.22,1.34)	1.07(1.02,1.13)
Cerebrovascular disease:		
Any cerebrovascular diagnoses	1.81(1.74,1.89)	1.13(1.09,1.18)
D. of stroke and TIA	1.69(1.63,1.76)	1.07(1.03,1.11)
Diabetes mellitus:		
D. of diabetes	1.96(1.86,2.07)	0.93(0.88,0.98)
Antidiabetics	1.49(1.43,1.54)	1.02(0.98,1.06)
Osteoporosis:		
D. of osteoporosis	1.70(1.61,1.80)	1.57(1.48,1.66)
Chronic lung disease:		
D. of chronic lung disease	2.53(2.42,2.65)	1.51(1.45,1.59)
Asthma/COPD medication	1.71(1.66,1.76)	1.40(1.36,1.44)
Oral steroids	2.17(2.09,2.26)	1.70(1.63,1.76)
Alcoholism-related disease:		
D. of alcoholism-related disease	2.34(2.09,2.62)	1.77(1.56,2.00)
Nutrition-related disease:		
D. of obesity	2.62(2.09,3.28)	2.11(1.66,2.69)
D. of undernutrition	2.46(2.00,3.02)	2.00(1.61,2.48)
Hypertrophia prostatae:		
D. of hypertrophia prostatae	1.45(1.38,1.52)	1.32(1.26,1.40)
Connective tissue disease:		
D. of connective tissue disease	1.71(1.60,1.84)	1.03(0.96,1.11)
Peptic ulcer disease:		
D. of peptic ulcer	2.14(2.00,2.28)	1.25(1.17,1.33)
Gastric acid related drugs	2.14(2.10,2.19)	1.88(1.84,1.92)
Low vision or blindness:		
D. of low vision or blindness	2.00(1.38,2.90)	1.55(1.05,2.28)
Hypercholesterolemia:		
D. of hypercholesterolemia	1.29(1.09,1.53)	1.09(0.91,1.31)
Chronic kidney disease:		
D. of chronic kidney disease	2.38(2.18,2.60)	0.97(0.89,1.06)
Anti-infective:		
Any antibiotic	1.47(1.46,1.49)	1.36(1.34,1.38)
Any D. of infections	2.15(2.09,2.21)	1.74(1.69,1.79)
Antibiotic systemic	1.56(1.54,1.59)	1.43(1.41,1.45)
D. of lower respiratory tract infections	2.25(2.17,2.34)	1.79(1.72,1.86)
D. of skin infections	1.95(1.83,2.08)	1.56(1.46,1.67)
Antibiotic topikal	1.45(1.38,1.53)	1.32(1.25,1.39)
D. of sepsis	2.71(2.49,2.94)	1.97(1.81,2.15)
D. of intestinal infectious diseases	2.40(2.24,2.57)	2.08(1.93,2.23)
Antibiotic Intestinale	3.60(3.23,4.00)	2.77(2.48,3.09)
D. of urinary tract infection	2.63(2.50,2.77)	2.15(2.04,2.27)
Symptoms not classified elsewhere:		
Symptoms not classified elsewhere	2.17(2.10,2.24)	1.78(1.72,1.84)
Observation:		
D. of observation	1.68(1.66,1.71)	1.39(1.37,1.41)

Axis (both panels): 1 2 3 4 5 — Prevalence Ratio with 95% CI

* Diagnosis of psychoses, schizophrenia, affective and personality disorders, †Dabigatran, rivaroxaban, apixaban, and edoxaban
All diagnoses are identified with a lookback period of 10 years from the index date and all medication use is identified with a lookback period of 12 months from the index date
D Diagnosis, PR Prevalence Ratio, CI Confidence Interval, CCI Charlson Comorbidity Index, RAS Renin Angiotensin System, NOAC Novel Oral Anticoagulants, TIA Transient Ischemic Attack

Cases n = 48,421

Supplementary Figure 10 Healthcare Utilization 12 Months Prior to Preventable Hospitalizations Due to Obstipation

	Sex and age adjusted PR	Sex, age, and CCI adjusted PR
ES consultation or home visit	1.00(0.99,1.00)	0.99(0.99,1.00)
GP consultation or home visit	1.01(1.01,1.01)	1.01(1.01,1.01)
No contacts	0.13(0.12,0.15)	0.20(0.18,0.22)
1-4 contacts	0.28(0.27,0.29)	0.36(0.34,0.37)
5+ contacts	1.20(1.19,1.20)	1.12(1.11,1.13)
Home visits	5.23(2.02,13.55)	4.49(1.70,11.81)
Preventive GP consultations and services	0.91(0.86,0.95)	0.89(0.84,0.94)
Social medicine-related GP consultations	2.22(2.13,2.32)	1.99(1.90,2.08)
GP conversational therapy	2.01(1.82,2.22)	1.76(1.58,1.95)
Reimbursement for chronically ill patients	2.65(2.27,3.09)	1.97(1.67,2.31)
Reimbursement for terminally ill patients	13.46(8.46,21.42)	6.65(4.15,10.65)
GP diagnosis and treatment of dementia	1.19(0.84,1.69)	1.21(0.85,1.73)
Influenza vaccine before index date	0.99(0.97,1.01)	0.93(0.91,0.95)
**Undergoing test at the GP within the last year:**		
Microbiological culture	1.62(1.57,1.68)	1.51(1.46,1.56)
Cytology test	0.81(0.71,0.93)	0.93(0.81,1.06)
Urine test	1.42(1.39,1.45)	1.35(1.32,1.37)
Strep-A test	1.23(1.12,1.36)	1.19(1.08,1.32)
Any blood test	1.05(1.03,1.06)	1.01(1.00,1.03)
C-reactive protein	1.38(1.34,1.41)	1.25(1.21,1.28)
Creatinin	1.22(1.08,1.38)	1.26(1.11,1.44)
Haemoglobin	1.23(1.21,1.26)	1.18(1.15,1.20)
Glucose	1.03(1.01,1.06)	1.02(1.00,1.05)
INR	1.08(1.02,1.15)	0.93(0.87,0.99)

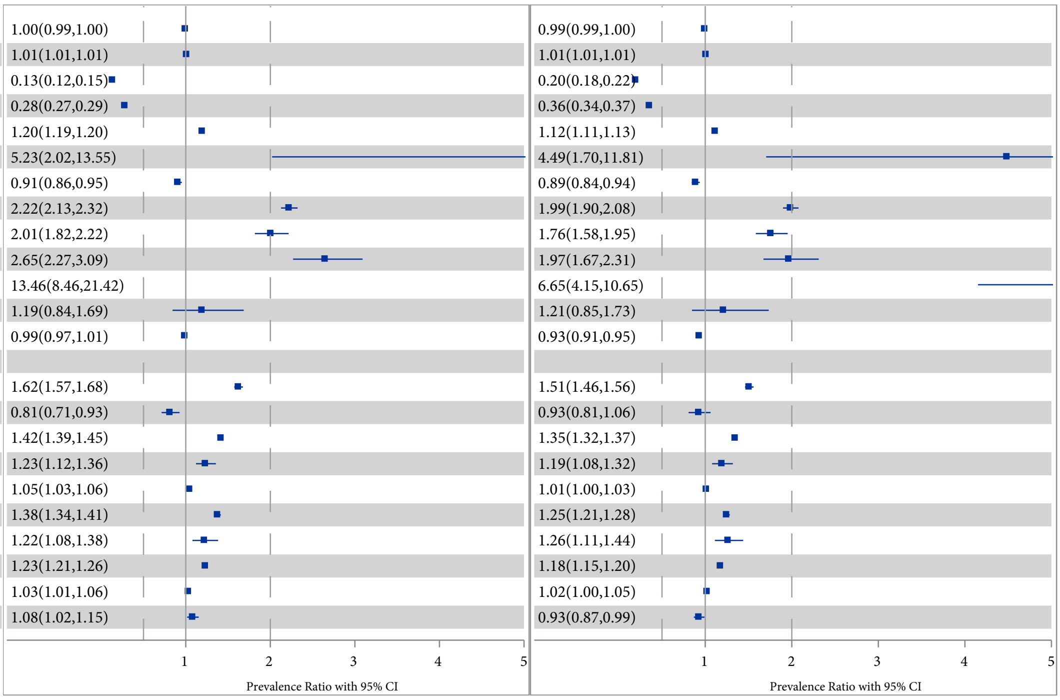

PR Prevalence Ratio, CI Confidence Interval, CCI Charlson Comorbidity Index, ES Doctor From the Emergency Service, GP General Practitioner, INR International Normalized Ratio

Cases n = 48,421

Supplementary Figure 11 Diagnoses and Medication Use Prior to Preventable Hospitalizations Due to Gastroenteritis

	Sex and age adjusted PR	Sex, age, and CCI adjusted PR
Psychiatric disease:		
D. of psychiatric disease*	2.14(1.84,2.47)	1.90(1.63,2.21)
Any nervous system drugs	1.35(1.33,1.36)	1.11(1.10,1.12)
Psycholeptic drugs	1.43(1.40,1.46)	1.36(1.33,1.39)
Antipsychotics	1.39(1.30,1.48)	1.32(1.23,1.41)
Anxiolytics	1.56(1.51,1.62)	1.48(1.43,1.54)
Hypnotics and sedatives	1.53(1.49,1.58)	1.44(1.39,1.48)
Benzodiazepine derivatives	1.56(1.50,1.61)	1.48(1.42,1.54)
Antidepressant drugs	1.75(1.69,1.80)	1.56(1.51,1.61)
Dementia:		
D. of dementia	1.39(1.25 , 1.55)	1.02(0.91 , 1.14)
Anti-dementia drugs	1.11(0.99 , 1.24)	0.99(0.88 , 1.11)
Cancer:		
D. of cancer	1.76(1.69,1.84)	0.90(0.87,0.93)
Cardiovascular disease:		
Any cardiovascular diagnoses	1.56(1.53,1.59)	1.31(1.28,1.34)
Ischemic heart disease	1.91(1.83,1.99)	1.62(1.56,1.69)
Heart failure	2.46(2.30,2.64)	1.48(1.38,1.58)
Heart valve disease	1.51(1.39,1.66)	1.27(1.16,1.39)
Atrial fibrilation	1.70(1.61,1.79)	1.46(1.38,1.54)
Peripheral artery disease	2.09(1.96,2.23)	1.22(1.14,1.29)
Deep vein thrombosis	1.73(1.54,1.94)	1.49(1.32,1.69)
Hypertension	1.77(1.67,1.89)	1.49(1.39,1.58)
Any cardiovascular medications	1.18(1.17,1.19)	1.07(1.06,1.07)
Antihypertensives except loop-diuretics	1.21(1.20,1.23)	1.15(1.14,1.17)
Agents acting on RAS	1.33(1.30,1.36)	1.21(1.18,1.24)
Other antihypertensives	1.29(1.27,1.32)	1.20(1.17,1.22)
Loop diuretics	1.83(1.78,1.89)	1.51(1.47,1.56)
Digoxin	1.67(1.58,1.76)	1.46(1.38,1.54)
Nitrates	1.78(1.70,1.87)	1.59(1.51,1.67)
Lipid modifying agents	1.29(1.26,1.33)	1.10(1.07,1.14)
NOAC†	1.72(1.53,1.92)	1.40(1.24,1.57)
Any thrombocyte-aggregation prophylaxis	1.36(1.34,1.39)	1.21(1.19,1.24)
Vitamin K antagonists	1.55(1.46,1.64)	1.32(1.24,1.40)
Cerebrovascular disease:		
Any cerebrovascular diagnoses	1.81(1.72,1.90)	1.18(1.12,1.24)
D. of stroke and TIA	1.74(1.66,1.82)	1.14(1.09,1.20)
Diabetes mellitus:		
D. of diabetes	2.38(2.23,2.55)	1.22(1.15,1.31)
Antidiabetics	1.72(1.64,1.80)	1.24(1.18,1.30)
Osteoporosis:		
D. of osteoporosis	1.54(1.44,1.64)	1.37(1.28,1.47)
Chronic lung disease:		
D. of chronic lung disease	2.23(2.10,2.36)	1.38(1.30,1.46)
Asthma/COPD medication	1.56(1.51,1.62)	1.31(1.26,1.36)
Oral steroids	2.24(2.14,2.34)	1.86(1.77,1.95)
Alcoholism-related disease:		
D. of alcoholism-related disease	2.77(2.38,3.23)	2.19(1.87,2.57)
Nutrition-related disease:		
D. of obesity	1.80(1.38,2.35)	1.41(1.07,1.86)
D. of undernutrition	2.14(1.67,2.73)	1.66(1.29,2.14)
Hypertrophia prostatae:		
D. of hypertrophia prostatae	1.21(1.13,1.30)	1.11(1.04,1.20)
Connective tissue disease:		
D. of connective tissue disease	2.12(1.96,2.30)	1.37(1.26,1.48)
Peptic ulcer disease:		
D. of peptic ulcer	2.41(2.23,2.61)	1.47(1.36,1.59)
Gastric acid related drugs	2.08(2.03,2.14)	1.84(1.79,1.88)
Low vision or blindness:		
D. of low vision or blindness	2.13(1.38,3.29)	1.79(1.14,2.79)
Hypercholesterolemia:		
D. of hypercholesterolemia	1.76(1.41,2.19)	1.46(1.16,1.84)
Chronic kidney disease:		
D. of chronic kidney disease	3.87(3.48,4.30)	1.68(1.51,1.87)
Anti-infective:		
Any antibiotic	1.45(1.43,1.47)	1.35(1.33,1.37)
Any D. of infections	2.46(2.38,2.56)	2.01(1.93,2.08)
Antibiotic systemic	1.53(1.50,1.55)	1.41(1.38,1.43)
D. of lower respiratory tract infections	2.43(2.32,2.55)	1.95(1.86,2.05)
D. of skin infections	1.97(1.82,2.14)	1.57(1.45,1.71)
Antibiotic topikal	1.45(1.36,1.54)	1.33(1.25,1.42)
D. of sepsis	3.18(2.86,3.54)	2.27(2.04,2.53)
D. of intestinal infectious diseases	4.90(4.42,5.43)	4.39(3.95,4.88)
Antibiotic Intestinale	3.06(2.69,3.48)	2.41(2.11,2.74)
D. of urinary tract infection	2.42(2.27,2.58)	2.00(1.88,2.14)
Symptoms not classified elsewhere:		
Symptoms not classified elsewhere	2.11(2.02,2.21)	1.79(1.71,1.87)
Observation:		
D. of observation	1.58(1.55,1.61)	1.33(1.30,1.36)

Axes: 1 2 3 4 5 — Prevalence Ratio with 95% CI (both panels)

* Diagnosis of psychoses, schizophrenia, affective and personality disorders, †Dabigatran, rivaroxaban, apixaban, and edoxaban

All diagnoses are identified with a lookback period of 10 years from the index date and all medication use is identified with a lookback period of 12 months from the index date

D Diagnosis, PR Prevalence Ratio, CI Confidence Interval, CCI Charlson Comorbidity Index, RAS Renin Angiotensin System, NOAC Novel Oral Anticoagulants, TIA Transient Ischemic Attack

Cases n = 31,832

Supplementary Figure 12 Healthcare Utilization 12 Months Prior to Preventable Hospitalizations Due to Gastroenteritis

	Sex and age adjusted PR	Sex, age, and CCI adjusted PR
ES consultation or home visit	1.00(0.99,1.00)	1.00(0.99,1.00)
GP consultation or home visit	1.01(1.01,1.01)	1.01(1.01,1.01)
No contacts	0.19(0.17,0.21)	0.26(0.23,0.29)
1-4 contacts	0.40(0.38,0.42)	0.49(0.47,0.52)
5+ contacts	1.16(1.15,1.17)	1.09(1.07,1.10)
Home visits	1.18(0.47,2.99)	1.11(0.43,2.87)
Preventive GP consultations and services	0.97(0.92,1.03)	0.97(0.91,1.03)
Social medicine-related GP consultations	1.97(1.87,2.08)	1.76(1.66,1.86)
GP conversational therapy	1.45(1.28,1.65)	1.32(1.16,1.50)
Reimbursement for chronically ill patients	1.99(1.62,2.43)	1.59(1.29,1.96)
Reimbursement for terminally ill patients	2.62(1.48,4.63)	1.52(0.85,2.74)
GP diagnosis and treatment of dementia	0.92(0.60,1.41)	0.91(0.59,1.40)
Influenza vaccine before index date	1.05(1.02,1.07)	0.99(0.96,1.01)
**Undergoing test at the GP within the last year:**		
Microbiological culture	1.39(1.34,1.45)	1.30(1.25,1.36)
Cytology test	0.82(0.70,0.95)	0.87(0.75,1.02)
Urine test	1.27(1.24,1.30)	1.21(1.18,1.24)
Strep-A test	1.28(1.14,1.45)	1.27(1.13,1.44)
Any blood test	1.06(1.04,1.08)	1.03(1.01,1.05)
C-reactive protein	1.42(1.37,1.47)	1.31(1.27,1.36)
Creatinin	1.37(1.19,1.58)	1.41(1.22,1.64)
Haemoglobin	1.22(1.19,1.25)	1.18(1.15,1.22)
Glucose	1.11(1.07,1.14)	1.09(1.06,1.12)
INR	1.30(1.20,1.40)	1.13(1.04,1.22)

Axis: 1 2 3 4 5 — Prevalence Ratio with 95% CI

PR Prevalence Ratio, CI Confidence Interval, CCI Charlson Comorbidity Index, ES Doctor From the Emergency Service, GP General Practitioner, INR International Normalized Ratio

Cases n = 31,832

Supplementary Figure 13 Diagnoses and Medication Use Prior to Preventable Hospitalizations Due to Anaemia

	Sex and age adjusted PR	Sex, age, and CCI adjusted PR
Psychiatric disease:		
D. of psychiatric disease*	1.48(1.24,1.77)	1.32(1.10,1.59)
Any nervous system drugs	1.29(1.27,1.31)	1.12(1.11,1.13)
Psycholeptic drugs	1.35(1.32,1.39)	1.30(1.27,1.34)
Antipsychotics	1.44(1.34,1.54)	1.40(1.30,1.50)
Anxiolytics	1.43(1.37,1.49)	1.38(1.33,1.45)
Hypnotics and sedatives	1.42(1.38,1.47)	1.35(1.30,1.39)
Benzodiazepine derivatives	1.41(1.35,1.48)	1.37(1.32,1.44)
Antidepressant drugs	1.57(1.51,1.63)	1.42(1.37,1.47)
Dementia:		
D. of dementia	1.16(1.03 , 1.30)	0.90(0.80 , 1.01)
Anti-dementia drugs	0.89(0.79 , 1.01)	0.82(0.72 , 0.93)
Cancer:		
D. of cancer	1.52(1.45,1.59)	0.88(0.85,0.91)
Cardiovascular disease:		
Any cardiovascular diagnoses	1.56(1.53,1.60)	1.29(1.26,1.32)
Ischemic heart disease	1.79(1.71,1.87)	1.55(1.48,1.62)
Heart failure	2.43(2.26,2.62)	1.59(1.48,1.71)
Heart valve disease	2.79(2.55,3.05)	2.47(2.26,2.71)
Atrial fibrilation	1.82(1.72,1.93)	1.58(1.49,1.68)
Peripheral artery disease	2.11(1.97,2.25)	1.31(1.23,1.40)
Deep vein thrombosis	1.54(1.35,1.75)	1.36(1.19,1.56)
Hypertension	1.58(1.47,1.69)	1.34(1.25,1.44)
Any cardiovascular medications	1.19(1.18,1.21)	1.08(1.07,1.09)
Antihypertensives except loop-diuretics	1.20(1.19,1.22)	1.14(1.13,1.16)
Agents acting on RAS	1.28(1.25,1.32)	1.17(1.14,1.20)
Other antihypertensives	1.25(1.23,1.28)	1.17(1.14,1.20)
Loop diuretics	1.94(1.88,2.00)	1.68(1.63,1.73)
Digoxin	1.72(1.63,1.82)	1.54(1.45,1.62)
Nitrates	1.83(1.74,1.93)	1.66(1.57,1.75)
Lipid modifying agents	1.30(1.26,1.34)	1.11(1.08,1.15)
NOAC†	3.02(2.71,3.37)	2.61(2.34,2.92)
Any thrombocyte-aggregation prophylaxis	1.50(1.47,1.54)	1.36(1.33,1.39)
Vitamin K antagonists	2.01(1.89,2.13)	1.73(1.62,1.84)
Cerebrovascular disease:		
Any cerebrovascular diagnoses	1.73(1.63,1.82)	1.19(1.13,1.26)
D. of stroke and TIA	1.67(1.58,1.75)	1.16(1.10,1.22)
Diabetes mellitus:		
D. of diabetes	2.20(2.04,2.37)	1.24(1.15,1.33)
Antidiabetics	1.87(1.78,1.96)	1.43(1.36,1.50)
Osteoporosis:		
D. of osteoporosis	1.21(1.11,1.31)	1.10(1.01,1.19)
Chronic lung disease:		
D. of chronic lung disease	1.98(1.86,2.11)	1.29(1.21,1.37)
Asthma/COPD medication	1.53(1.47,1.60)	1.32(1.26,1.38)
Oral steroids	1.70(1.62,1.80)	1.44(1.36,1.52)
Alcoholism-related disease:		
D. of alcoholism-related disease	2.92(2.51,3.41)	2.23(1.90,2.61)
Nutrition-related disease:		
D. of obesity	1.97(1.41,2.74)	1.65(1.17,2.32)
D. of undernutrition	2.09(1.59,2.75)	1.59(1.20,2.11)
Hypertrophia prostatae:		
D. of hypertrophia prostatae	1.10(1.02,1.19)	1.01(0.94,1.10)
Connective tissue disease:		
D. of connective tissue disease	1.65(1.50,1.82)	1.12(1.02,1.24)
Peptic ulcer disease:		
D. of peptic ulcer	2.67(2.47,2.90)	1.78(1.64,1.93)
Gastric acid related drugs	2.02(1.96,2.07)	1.79(1.75,1.84)
Low vision or blindness:		
D. of low vision or blindness	0.84(0.52,1.35)	0.77(0.48,1.26)
Hypercholesterolemia:		
D. of hypercholesterolemia	1.40(1.09,1.79)	1.05(0.81,1.36)
Chronic kidney disease:		
D. of chronic kidney disease	2.57(2.27,2.90)	1.27(1.12,1.43)
Anti-infective:		
Any antibiotic	1.29(1.27,1.31)	1.20(1.18,1.23)
Any D. of infections	1.77(1.70,1.84)	1.47(1.41,1.53)
Antibiotic systemic	1.36(1.33,1.39)	1.26(1.23,1.28)
D. of lower respiratory tract infections	1.87(1.78,1.97)	1.54(1.46,1.62)
D. of skin infections	1.62(1.48,1.77)	1.35(1.23,1.48)
Antibiotic topikal	1.25(1.16,1.34)	1.14(1.06,1.23)
D. of sepsis	2.25(1.99,2.54)	1.75(1.54,1.98)
D. of intestinal infectious diseases	1.60(1.45,1.77)	1.39(1.26,1.54)
Antibiotic Intestinale	2.60(2.25,3.02)	2.12(1.83,2.47)
D. of urinary tract infection	1.60(1.48,1.72)	1.36(1.27,1.47)
Symptoms not classified elsewhere:		
Symptoms not classified elsewhere	1.75(1.67,1.84)	1.51(1.44,1.59)
Observation:		
D. of observation	1.47(1.44,1.50)	1.25(1.23,1.28)

Prevalence Ratio with 95% CI (axis 1–5) | Prevalence Ratio with 95% CI (axis 1–5)

* Diagnosis of psychoses, schizophrenia, affective and personality disorders, †Dabigatran, rivaroxaban, apixaban, and edoxaban
All diagnoses are identified with a lookback period of 10 years from the index date and all medication use is identified with a lookback period of 12 months from the index date
D Diagnosis, PR Prevalence Ratio, CI Confidence Interval, CCI Charlson Comorbidity Index, RAS Renin Angiotensin System, NOAC Novel Oral Anticoagulants, TIA Transient Ischemic Attack

Cases n = 25,603

Supplementary Figure 14 Healthcare Utilization 12 Months Prior to Preventable Hospitalizations Due to Anaemia

	Sex and age adjusted PR	Sex, age, and CCI adjusted PR
ES consultation or home visit	1.00(0.99,1.00)	0.99(0.98,1.00)
GP consultation or home visit	1.01(1.01,1.01)	1.01(1.00,1.01)
No contacts	0.27(0.25,0.31)	0.37(0.33,0.41)
1-4 contacts	0.39(0.37,0.42)	0.48(0.45,0.51)
5+ contacts	1.15(1.14,1.16)	1.08(1.07,1.10)
Home visits	0.68(0.22,2.14)	0.69(0.22,2.20)
Preventive GP consultations and services	1.03(0.96,1.10)	1.00(0.93,1.07)
Social medicine-related GP consultations	1.82(1.72,1.93)	1.66(1.57,1.76)
GP conversational therapy	1.24(1.06,1.44)	1.12(0.96,1.31)
Reimbursement for chronically ill patients	2.12(1.69,2.64)	1.61(1.28,2.02)
Reimbursement for terminally ill patients	3.76(2.18,6.50)	2.18(1.25,3.81)
GP diagnosis and treatment of dementia	1.09(0.71,1.68)	1.10(0.71,1.69)
Influenza vaccine before index date	0.98(0.95,1.00)	0.92(0.90,0.95)
**Undergoing test at the GP within the last year:**		
Microbiological culture	1.26(1.20,1.31)	1.18(1.13,1.24)
Cytology test	0.59(0.48,0.73)	0.63(0.51,0.78)
Urine test	1.20(1.16,1.23)	1.14(1.11,1.18)
Strep-A test	0.96(0.84,1.10)	0.92(0.80,1.07)
Any blood test	1.24(1.21,1.26)	1.20(1.17,1.22)
C-reactive protein	1.41(1.36,1.47)	1.30(1.25,1.36)
Creatinin	1.49(1.26,1.76)	1.51(1.27,1.78)
Haemoglobin	1.78(1.74,1.83)	1.73(1.68,1.77)
Glucose	1.23(1.19,1.27)	1.21(1.17,1.24)
INR	1.89(1.75,2.04)	1.68(1.55,1.82)

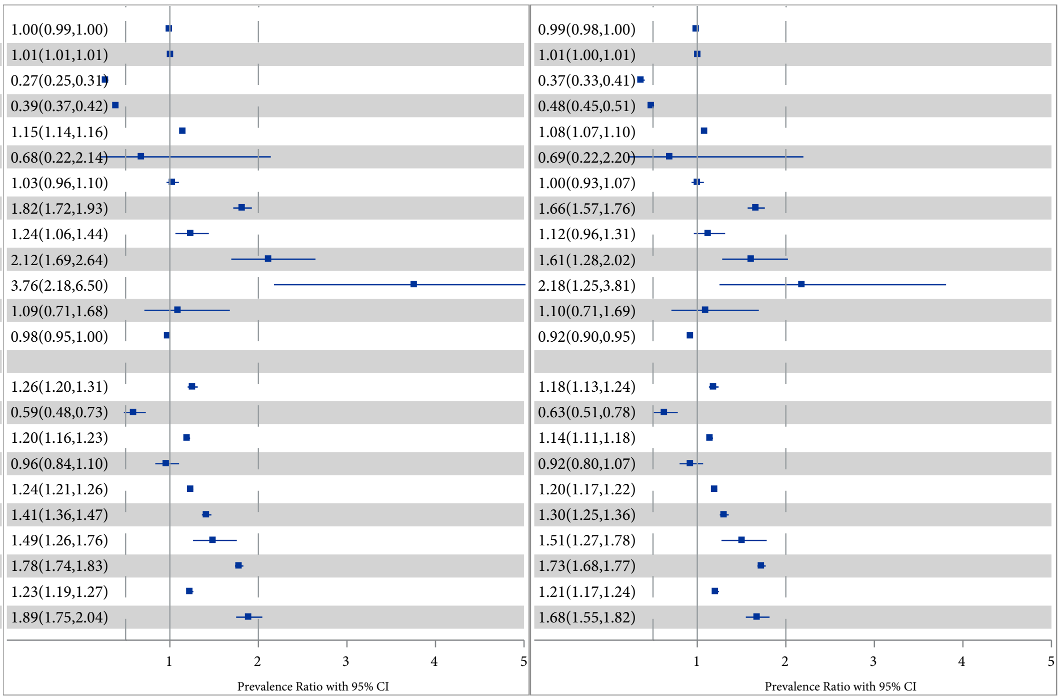

PR Prevalence Ratio, CI Confidence Interval, CCI Charlson Comorbidity Index, ES Doctor From the Emergency Service, GP General Practitioner, INR International Normalized Ratio

Cases n = 25,603

Supplementary Figure 15 Diagnoses and Medication Use Prior to Preventable Hospitalizations Due to Social Causes

	Sex and age adjusted PR	Sex, age, and CCI adjusted PR
Psychiatric disease:		
D. of psychiatric disease*	3.06(2.56,3.66)	2.93(2.43,3.53)
Any nervous system drugs	1.41(1.38,1.43)	1.29(1.27,1.31)
Psycholeptic drugs	1.52(1.48,1.56)	1.40(1.37,1.44)
Antipsychotics	2.21(2.06,2.37)	2.17(2.02,2.34)
Anxiolytics	1.81(1.74,1.89)	1.67(1.60,1.75)
Hypnotics and sedatives	1.54(1.49,1.60)	1.38(1.33,1.43)
Benzodiazepine derivatives	1.82(1.75,1.90)	1.68(1.61,1.76)
Antidepressant drugs	1.97(1.89,2.06)	1.69(1.62,1.77)
Dementia:		
D. of dementia	3.06(2.69,3.48)	2.25(1.97,2.57)
Anti-dementia drugs	1.64(1.39,1.93)	1.54(1.29,1.83)
Cancer:		
D. of cancer	3.19(3.05,3.34)	1.42(1.36,1.48)
Cardiovascular disease:		
Any cardiovascular diagnoses	1.51(1.47,1.56)	1.13(1.10,1.16)
Ischemic heart disease	1.37(1.29,1.46)	1.06(0.99,1.13)
Heart failure	2.39(2.18,2.63)	1.35(1.22,1.48)
Heart valve disease	1.07(0.92,1.24)	0.80(0.68,0.94)
Atrial fibrillation	1.58(1.46,1.71)	1.28(1.18,1.39)
Peripheral artery disease	1.60(1.46,1.75)	0.76(0.69,0.83)
Deep vein thrombosis	2.15(1.81,2.56)	1.67(1.39,2.01)
Hypertension	1.53(1.40,1.69)	1.16(1.05,1.28)
Any cardiovascular medications	1.11(1.09,1.13)	1.02(1.01,1.04)
Antihypertensives except loop-diuretics	1.09(1.07,1.11)	1.00(0.98,1.02)
Agents acting on RAS	0.94(0.91,0.98)	0.77(0.74,0.80)
Other antihypertensives	1.02(0.99,1.06)	0.90(0.87,0.93)
Loop diuretics	1.47(1.42,1.52)	1.20(1.16,1.25)
Digoxin	1.53(1.45,1.62)	1.34(1.26,1.41)
Nitrates	1.16(1.09,1.23)	0.97(0.91,1.04)
Lipid modifying agents	0.92(0.87,0.97)	0.67(0.63,0.72)
NOAC†	1.68(1.35,2.09)	1.38(1.10,1.74)
Any thrombocyte-aggregation prophylaxis	1.18(1.14,1.21)	1.00(0.97,1.03)
Vitamin K antagonists	1.34(1.22,1.46)	1.02(0.92,1.12)
Cerebrovascular disease:		
Any cerebrovascular diagnoses	2.23(2.09,2.39)	1.34(1.25,1.43)
D. of stroke and TIA	2.07(1.94,2.20)	1.25(1.17,1.33)
Diabetes mellitus:		
D. of diabetes	1.93(1.75,2.13)	0.84(0.76,0.93)
Antidiabetics	1.34(1.25,1.43)	0.85(0.79,0.91)
Osteoporosis:		
D. of osteoporosis	1.76(1.59,1.96)	1.59(1.42,1.77)
Chronic lung disease:		
D. of chronic lung disease	1.98(1.83,2.15)	1.02(0.94,1.11)
Asthma/COPD medication	1.34(1.27,1.41)	1.00(0.95,1.06)
Oral steroids	2.28(2.15,2.42)	1.50(1.41,1.60)
Alcoholism-related disease:		
D. of alcoholism-related disease	4.87(4.03,5.89)	5.23(4.28,6.40)
Nutrition-related disease:		
D. of obesity	2.34(1.49,3.67)	2.08(1.27,3.41)
D. of undernutrition	2.31(1.77,3.02)	1.90(1.44,2.52)
Hypertrophia prostatae:		
D. of hypertrophia prostatae	1.28(1.17,1.39)	1.11(1.02,1.22)
Connective tissue disease:		
D. of connective tissue disease	1.51(1.33,1.71)	0.79(0.69,0.90)
Peptic ulcer disease:		
D. of peptic ulcer	2.11(1.90,2.35)	1.13(1.02,1.26)
Gastric acid related drugs	1.77(1.71,1.84)	1.39(1.34,1.44)
Low vision or blindness:		
D. of low vision or blindness	1.47(1.04,2.08)	1.11(0.77,1.60)
Hypercholesterolemia:		
D. of hypercholesterolemia	0.75(0.49,1.16)	0.46(0.29,0.75)
Chronic kidney disease:		
D. of chronic kidney disease	2.41(2.02,2.87)	0.78(0.65,0.93)
Anti-infective:		
Any antibiotic	1.29(1.26,1.31)	1.14(1.12,1.17)
Any D. of infections	2.25(2.14,2.36)	1.73(1.64,1.82)
Antibiotic systemic	1.33(1.30,1.36)	1.16(1.13,1.19)
D. of lower respiratory tract infections	2.37(2.23,2.53)	1.79(1.67,1.91)
D. of skin infections	1.89(1.68,2.12)	1.48(1.31,1.68)
Antibiotic topikal	1.14(1.05,1.24)	1.01(0.92,1.10)
D. of sepsis	3.20(2.71,3.78)	2.19(1.84,2.61)
D. of intestinal infectious diseases	1.97(1.79,2.18)	1.71(1.54,1.90)
Antibiotic Intestinale	3.47(2.90,4.15)	2.27(1.87,2.74)
D. of urinary tract infection	2.79(2.54,3.05)	2.31(2.10,2.54)
Symptoms not classified elsewhere:		
Symptoms not classified elsewhere	2.52(2.37,2.69)	1.96(1.83,2.09)
Observation:		
D. of observation	1.95(1.89,2.01)	1.48(1.43,1.53)

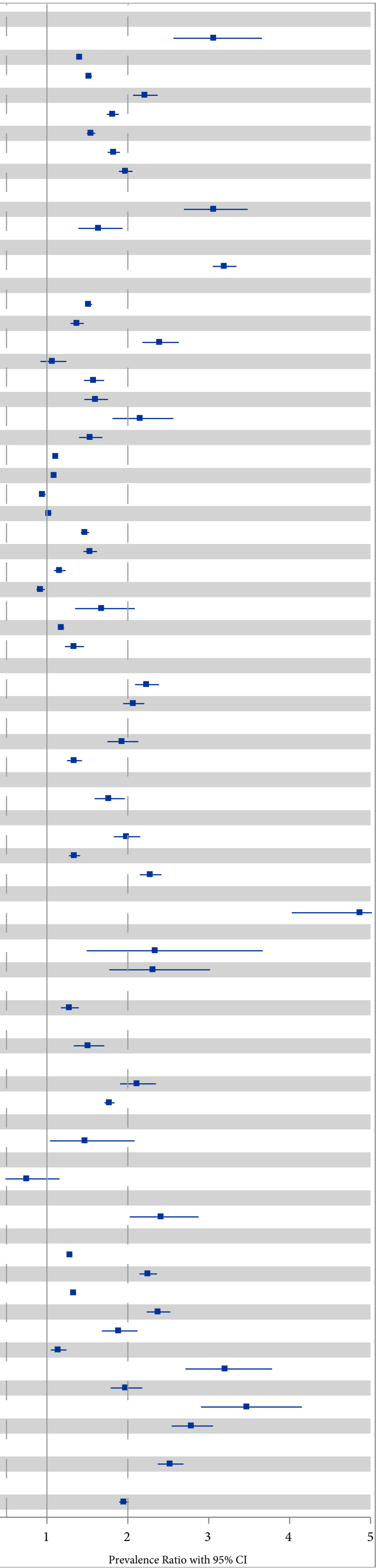

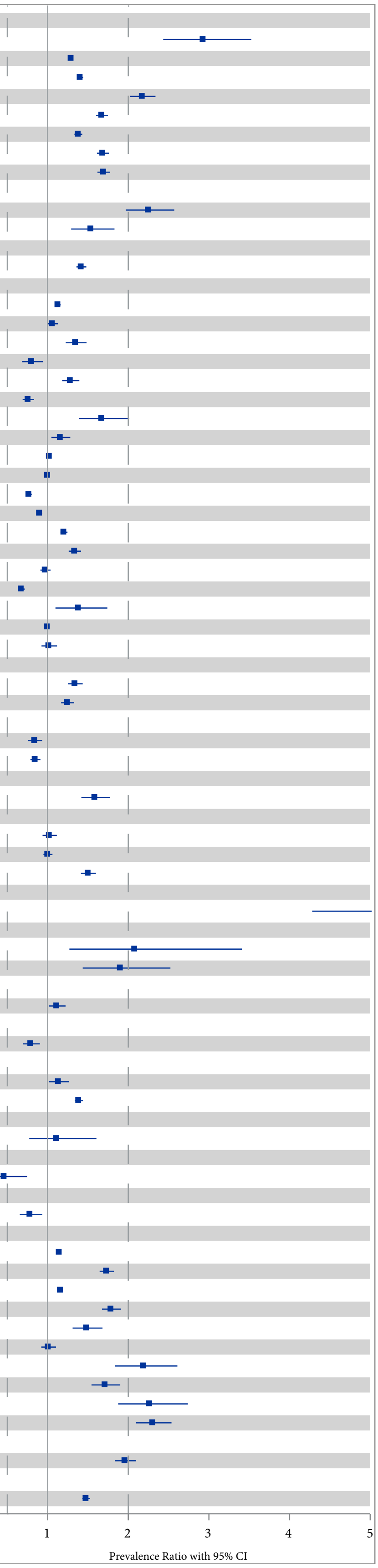

* Diagnosis of psychoses, schizophrenia, affective and personality disorders, †Dabigatran, rivaroxaban, apixaban, and edoxaban
All diagnoses are identified with a lookback period of 10 years from the index date and all medication use is identified with a lookback period of 12 months from the index date
D Diagnosis, PR Prevalence Ratio, CI Confidence Interval, CCI Charlson Comorbidity Index, RAS Renin Angiotensin System, NOAC Novel Oral Anticoagulants, TIA Transient Ischemic Attack

Cases n = 19,117

Supplementary Figure 16 Healthcare Utilization 12 Months Prior to Preventable Hospitalizations Due to Social Causes

	Sex and age adjusted PR	Sex, age, and CCI adjusted PR
ES consultation or home visit	0.91(0.90,0.92)	0.91(0.89,0.92)
GP consultation or home visit	1.00(1.00,1.00)	1.00(1.00,1.00)
No contacts	0.39(0.36,0.43)	0.61(0.55,0.67)
1-4 contacts	0.49(0.46,0.52)	0.65(0.62,0.70)
5+ contacts	1.15(1.14,1.16)	1.08(1.06,1.09)
Home visits	2.25(1.11,4.55)	1.45(0.69,3.04)
Preventive GP consultations and services	0.87(0.79,0.96)	0.77(0.69,0.86)
Social medicine-related GP consultations	2.86(2.70,3.04)	2.61(2.46,2.77)
GP conversational therapy	2.90(2.42,3.48)	2.07(1.70,2.52)
Reimbursement for chronically ill patients	4.00(2.96,5.40)	2.23(1.62,3.07)
Reimbursement for terminally ill patients	70.73(29.28,170.86)	30.46(12.55,73.96)
GP diagnosis and treatment of dementia	1.90(1.14,3.17)	1.74(1.02,2.97)
Influenza vaccine before index date	0.83(0.79,0.86)	0.70(0.67,0.74)
**Undergoing test at the GP within the last year:**		
Microbiological culture	1.21(1.15,1.28)	1.08(1.02,1.15)
Cytology test	0.52(0.39,0.70)	0.54(0.39,0.74)
Urine test	1.10(1.06,1.14)	1.01(0.98,1.05)
Strep-A test	0.81(0.68,0.98)	0.70(0.57,0.86)
Any blood test	0.82(0.79,0.85)	0.75(0.72,0.78)
C-reactive protein	1.10(1.03,1.18)	0.88(0.82,0.95)
Creatinin	1.55(1.31,1.82)	1.66(1.40,1.98)
Haemoglobin	0.99(0.95,1.02)	0.88(0.85,0.92)
Glucose	0.88(0.84,0.92)	0.82(0.78,0.86)
INR	1.05(0.91,1.21)	0.82(0.70,0.95)

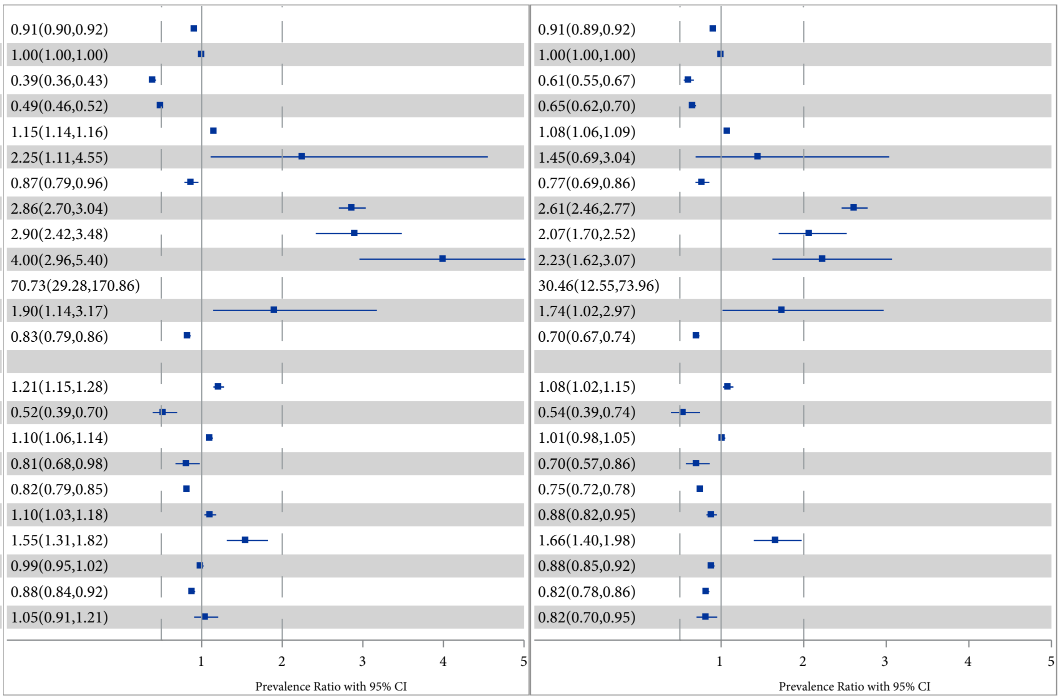

PR Prevalence Ratio, CI Confidence Interval, CCI Charlson Comorbidity Index, ES Doctor From the Emergency Service, GP General Practitioner, INR International Normalized Ratio

Cases n = 19,117

Supplementary Figure 17 Diagnoses and Medication Use Prior to Preventable Hospitalizations Due to Pressure Ulcer

	Sex and age adjusted PR	Sex, age, and CCI adjusted PR
Psychiatric disease:		
D. of psychiatric disease*	3.32(2.34,4.69)	3.28(2.29,4.69)
Any nervous system drugs	1.56(1.52,1.60)	1.16(1.14,1.18)
Psycholeptic drugs	1.59(1.52,1.66)	1.53(1.46,1.61)
Antipsychotics	3.46(3.05,3.93)	3.58(3.14,4.08)
Anxiolytics	1.64(1.51,1.77)	1.61(1.48,1.75)
Hypnotics and sedatives	1.50(1.40,1.60)	1.39(1.30,1.49)
Benzodiazepine derivatives	1.63(1.50,1.76)	1.61(1.48,1.75)
Antidepressant drugs	2.18(2.03,2.34)	1.98(1.84,2.13)
Dementia:		
D. of dementia	2.81(2.27,3.48)	2.00(1.60,2.49)
Anti-dementia drugs	1.52(1.22 , 1.91)	1.37(1.07 , 1.75)
Cancer:		
D. of cancer	1.58(1.43,1.73)	0.75(0.69,0.81)
Cardiovascular disease:		
Any cardiovascular diagnoses	1.73(1.66,1.80)	1.32(1.26,1.38)
Ischemic heart disease	1.19(1.07,1.32)	0.89(0.80,0.99)
Heart failure	2.41(2.05,2.82)	1.25(1.06,1.47)
Heart valve disease	1.38(1.10,1.73)	1.11(0.87,1.42)
Atrial fibrilation	1.71(1.51,1.94)	1.35(1.18,1.54)
Peripheral artery disease	3.99(3.49,4.55)	2.05(1.79,2.35)
Deep vein thrombosis	2.41(1.88,3.08)	1.99(1.54,2.57)
Hypertension	1.37(1.19,1.59)	1.07(0.91,1.25)
Any cardiovascular medications	1.19(1.17,1.22)	1.06(1.04,1.08)
Antihypertensives except loop-diuretics	1.09(1.06,1.12)	1.00(0.97,1.03)
Agents acting on RAS	1.03(0.97,1.09)	0.86(0.81,0.91)
Other antihypertensives	0.97(0.92,1.02)	0.85(0.81,0.89)
Loop diuretics	2.54(2.39,2.69)	2.29(2.12,2.48)
Digoxin	1.69(1.52,1.88)	1.40(1.25,1.56)
Nitrates	1.03(0.91,1.16)	0.86(0.76,0.98)
Lipid modifying agents	0.98(0.91,1.05)	0.74(0.68,0.79)
NOAC†	2.02(1.58,2.59)	1.58(1.22,2.05)
Any thrombocyte-aggregation prophylaxis	1.36(1.30,1.43)	1.16(1.10,1.22)
Vitamin K antagonists	1.57(1.37,1.79)	1.21(1.05,1.39)
Cerebrovascular disease:		
Any cerebrovascular diagnoses	2.79(2.51,3.10)	1.74(1.55,1.94)
D. of stroke and TIA	2.32(2.10,2.57)	1.45(1.31,1.61)
Diabetes mellitus:		
D. of diabetes	3.62(3.15,4.16)	1.54(1.34,1.76)
Antidiabetics	2.35(2.13,2.59)	1.45(1.31,1.61)
Osteoporosis:		
D. of osteoporosis	1.59(1.36,1.86)	1.42(1.20,1.67)
Chronic lung disease:		
D. of chronic lung disease	1.56(1.36,1.80)	0.83(0.72,0.96)
Asthma/COPD medication	1.06(0.97,1.17)	0.81(0.74,0.90)
Oral steroids	1.65(1.48,1.85)	1.29(1.15,1.46)
Alcoholism-related disease:		
D. of alcoholism-related disease	4.72(3.29,6.78)	4.44(3.03,6.50)
Nutrition-related disease:		
D. of obesity	5.77(2.85,11.70)	4.30(2.05,9.02)
D. of undernutrition	2.00(1.24,3.23)	1.64(0.99,2.71)
Hypertrophia prostatae:		
D. of hypertrophia prostatae	1.32(1.12,1.55)	1.17(0.98,1.39)
Connective tissue disease:		
D. of connective tissue disease	2.17(1.77,2.66)	1.28(1.04,1.59)
Peptic ulcer disease:		
D. of peptic ulcer	1.95(1.64,2.32)	1.06(0.88,1.26)
Gastric acid related drugs	1.95(1.84,2.07)	1.68(1.58,1.78)
Low vision or blindness:		
D. of low vision or blindness	1.45(0.68,3.13)	0.99(0.44,2.20)
Hypercholesterolemia:		
D. of hypercholesterolemia	0.83(0.42,1.65)	0.50(0.24,1.07)
Chronic kidney disease:		
D. of chronic kidney disease	4.32(3.31,5.65)	1.43(1.10,1.86)
Anti-infective:		
Any antibiotic	1.85(1.79,1.91)	1.75(1.69,1.80)
Any D. of infections	2.94(2.73,3.18)	2.38(2.20,2.58)
Antibiotic systemic	2.08(2.00,2.15)	1.94(1.87,2.01)
D. of lower respiratory tract infections	2.74(2.47,3.03)	2.16(1.94,2.41)
D. of skin infections	4.48(3.81,5.27)	3.62(3.06,4.29)
Antibiotic topikal	1.93(1.70,2.19)	1.74(1.52,1.99)
D. of sepsis	7.47(5.98,9.34)	5.65(4.48,7.11)
D. of intestinal infectious diseases	1.78(1.46,2.17)	1.50(1.22,1.85)
Antibiotic Intestinale	3.70(2.77,4.95)	3.04(2.25,4.11)
D. of urinary tract infection	4.83(4.18,5.58)	4.01(3.46,4.66)
Symptoms not classified elsewhere:		
Symptoms not classified elsewhere	2.15(1.94,2.38)	1.70(1.52,1.89)
Observation:		
D. of observation	1.57(1.50,1.64)	1.26(1.20,1.32)

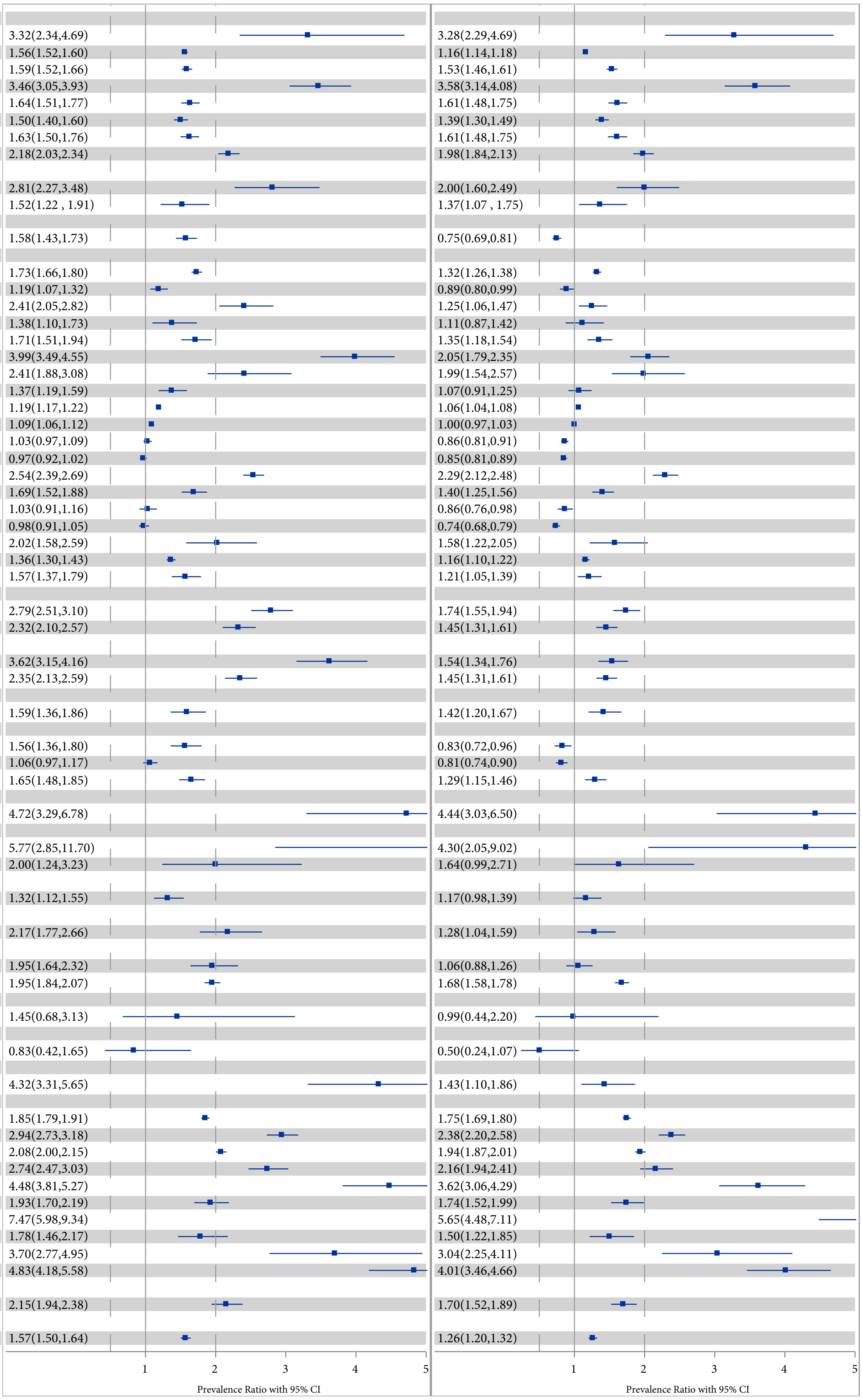

* Diagnosis of psychoses, schizophrenia, affective and personality disorders, †Dabigatran, rivaroxaban, apixaban, and edoxaban

All diagnoses are identified with a lookback period of 10 years from the index date and all medication use is identified with a lookback period of 12 months from the index date

D Diagnosis, PR Prevalence Ratio, CI Confidence Interval, CCI Charlson Comorbidity Index, RAS Renin Angiotensin System, NOAC Novel Oral Anticoagulants, TIA Transient Ischemic Attack

Cases n = 6,137

Supplementary Figure 18 Healthcare Utilization 12 Months Prior to Preventable Hospitalizations Due to Pressure Ulcer

	Sex and age adjusted PR	Sex, age, and CCI adjusted PR
ES consultation or home visit	0.89(0.87,0.91)	0.89(0.87,0.91)
GP consultation or home visit	1.01(1.00,1.01)	1.01(1.00,1.01)
No contacts	0.25(0.20,0.31)	0.40(0.32,0.50)
1-4 contacts	0.24(0.21,0.28)	0.32(0.28,0.38)
5+ contacts	1.19(1.17,1.20)	1.10(1.08,1.13)
Home visits	1.039E11(1.039E11,1.	9.902E10(9.902E10,9.
Preventive GP consultations and services	0.76(0.65,0.88)	0.72(0.61,0.85)
Social medicine-related GP consultations	4.14(3.72,4.60)	3.95(3.54,4.41)
GP conversational therapy	0.93(0.67,1.30)	0.82(0.58,1.17)
Reimbursement for chronically ill patients	2.73(1.74,4.29)	2.02(1.25,3.25)
Reimbursement for terminally ill patients	10.79(2.54,45.72)	6.31(1.45,27.52)
GP diagnosis and treatment of dementia	0.94(0.30,2.92)	1.09(0.33,3.56)
Influenza vaccine before index date	0.85(0.80,0.90)	0.79(0.74,0.84)
**Undergoing test at the GP within the last year:**		
Microbiological culture	1.81(1.66,1.98)	1.69(1.54,1.85)
Cytology test	0.33(0.20,0.54)	0.31(0.18,0.52)
Urine test	1.37(1.30,1.45)	1.29(1.22,1.37)
Strep-A test	0.37(0.24,0.55)	0.40(0.26,0.62)
Any blood test	0.79(0.75,0.84)	0.76(0.72,0.80)
C-reactive protein	0.82(0.75,0.90)	0.73(0.66,0.81)
Creatinin	0.87(0.59,1.28)	0.87(0.58,1.31)
Haemoglobin	0.88(0.82,0.94)	0.84(0.78,0.90)
Glucose	0.82(0.76,0.88)	0.79(0.73,0.86)
INR	1.35(1.11,1.63)	1.07(0.87,1.31)

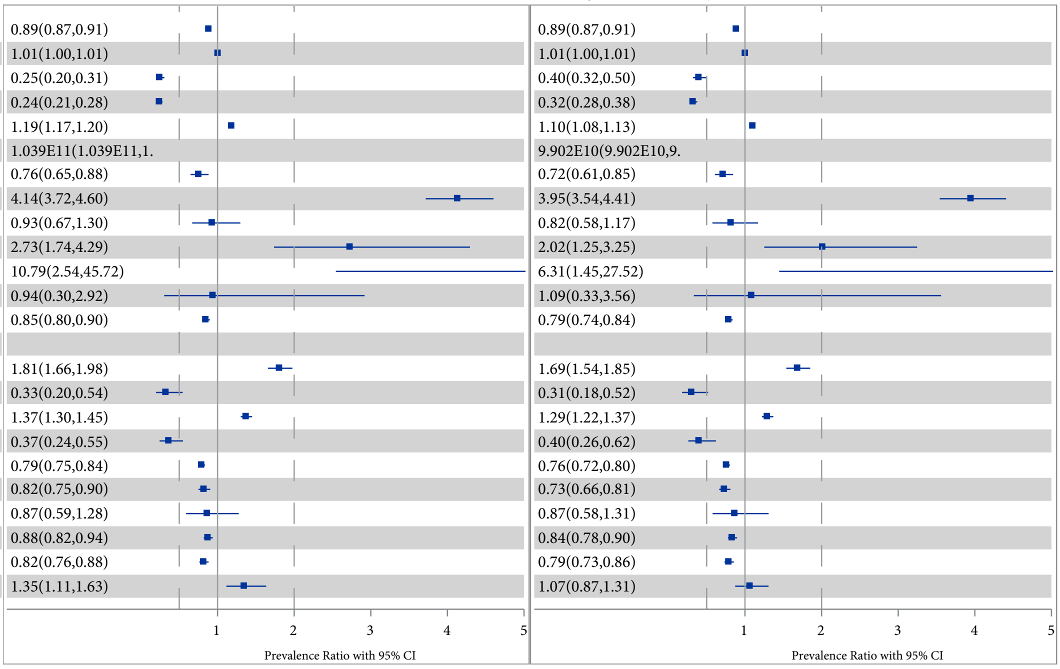

Cases n = 6,137

PR Prevalence Ratio, CI Confidence Interval, CCI Charlson Comorbidity Index, ES Doctor From the Emergency Service, GP General Practitioner, INR International Normalized Ratio
